# Photoredox catalysis enabling decarboxylative radical cyclization of γ,γ-dimethylallyltryptophan (DMAT) derivatives: formal synthesis of 6,7-secoagroclavine

**DOI:** 10.3762/bjoc.19.70

**Published:** 2023-06-26

**Authors:** Alessio Regni, Francesca Bartoccini, Giovanni Piersanti

**Affiliations:** 1 Department of Biomolecular Sciences, University of Urbino, Carlo Bo Piazza Rinascimento 6, 61029 Urbino, PU, Italy

**Keywords:** decarboxylative cyclization, DMAT, ergot alkaloids, photoredox catalysis, radicals

## Abstract

An unusual photoredox-catalyzed radical decarboxylative cyclization cascade reaction of γ,γ-dimethylallyltryptophan (DMAT) derivatives containing unactivated alkene moieties has been developed, providing green and efficient access to various six-, seven-, and eight-membered ring 3,4-fused tricyclic indoles. This type of cyclization, which was hitherto very difficult to comprehend in ergot biosynthesis and to accomplish by more conventional procedures, enables the synthesis of ergot alkaloid precursors. In addition, this work describes a mild, environmentally friendly method to activate, reductively and oxidatively, natural carboxylic acids for decarboxylative C–C bond formation by exploiting the same photocatalyst.

## Introduction

Visible-light photoredox catalysis is rapidly changing the way organic chemists approach the design and synthesis of molecules by offering new synthetic disconnection opportunities that are usually more convergent, enabling a more diverse chemical space in a rapid fashion [1–15]. Currently, increasing numbers of synthetic chemists are developing photocatalytic processes to make molecules efficiently and in an environmentally friendly manner due to their intrinsic mildness and broad substrate compatibility [16–20]. This transformative synthetic tool often utilizes direct single-electron transfer (SET) between an electronically excited photoredox catalyst and an organic substrate, resulting in oxidation or reduction, to easily generate reactive open-shell radical species and/or intermediates. The substrate is consequently activated for bond cleavage, atom abstraction, or nucleophilic or electrophilic attack. After quenching, the oxidized or reduced photocatalyst regains or loses an electron to return to the starting ground state catalyst [21–26].

While early research has focused on methods for the functionalization of relatively simple hydrocarbons [27–30], developments in photoredox catalysis have gained traction recently as a viable strategy for the total synthesis of natural products [31–33], modification of biomacromolecules [34], and relatively complex pharmaceutical agents [35–38]. Photocatalysis tremendously enriches the synthetic compound library, providing a precious alternative to directly modify abundant natural substrates, including biomass, which usually requires a multistep process in conventional chemical synthesis [39–41]. Among the various widely available and abundant substrates for photocatalyzed reactions, natural and unnatural α-amino acids play a very important role, given their paramount importance across several chemistry fields as well as their ability to participate in either redox step of the catalytic cycle [42–45]. For example, the main use of α-amino acids in syntheses via photoredox catalysis is as readily available precursors of regioselective α-amino radicals by decarboxylative transformations, by oxidation of the carboxylate anion and/or reduction of the corresponding *N*-hydroxyphthalimide- (NHPI)-derived redox-active ester, although it destroys their stereochemical information [46–51]. In addition, the side-chains of aromatic amino acids (mainly electron-rich tryptophan and tyrosine) can be selectively targeted by photoredox catalysis to enable unprecedented modification of the amino acid. In this context, it is worth mentioning that the single-electron oxidation of the indole moiety in tryptophan provides the radical cation, which enables selective C-radical generation at the weaker benzylic position via a sequential electron transfer–proton transfer (ET/PT) [52–59].

**Figure 1:**
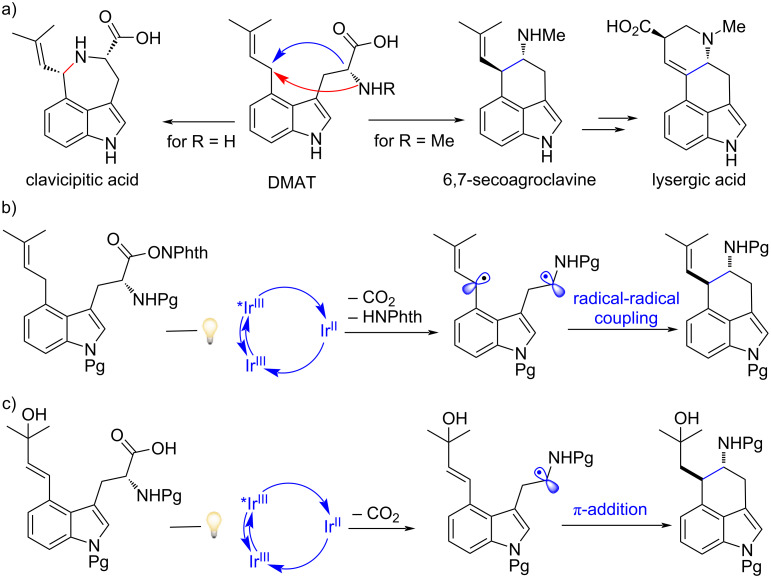
(a) Transformations of DMAT to different classes of ergot alkaloids. (b) and (c) Strategies for the photoredox-catalyzed radical decarboxylative cyclization cascade reaction of DMAT derivatives (this work).

With our ongoing interest of establishing new methods for the asymmetric synthesis of nonproteinogenic tryptophan derivatives as well as their associated indole alkaloid natural products [60–67], we became fascinated in exploring whether photoredox catalysis could be applied for the activation of such unnatural amino acids to expedite the development of completely new synthetic pathways. In particular, 4-dimethylallyltryptophan (DMAT) is of interest for the following reasons: 1) it functions as the central intermediate in the biosynthetic pathways leading to numerous prenylated indole alkaloids, such as ergot alkaloids in normal biosynthesis and clavicipitic acid in derailment biosynthesis [68–71]; and 2) the mechanism of the fundamental central C-ring formation of all ergot alkaloids, specifically the decarboxylative cyclization of DMAT, is still a puzzle even though a radical mechanism has been proposed (Figure 1a) [72–73].

## Results and Discussion

Herein, we propose that visible light irradiation of the cationic iridium photocatalyst Ir[dF(CF_3_)ppy]_2_(dtbbpy)PF_6_ (*E*_1/2_^*III/II^ = +1.21 V, *E*_1/2_^III/II^ = −1.37; *E*_1/2_^IV/*III^ = −0.89, *E*_1/2_^ IV/III^ = +1.69 V) [74] would permit efficient radical generation and C(sp^3^)–C(sp^3^) bond formation either by challenging selective radical–radical cross-coupling or by radical addition to a π-bond, enabling a rare example of intramolecular decarboxylative cyclization with the formation of the 3,4-fused indole carbocycle rings (Figure 1b,c). In detail, the photocatalytic strategy for accessing the two C(sp^3^) radicals of DMAT derivatives envision the formation of a relatively stabilized allylic-benzylic carbon-centered radical by proton transfer from the oxidized indole radical cation [75], generated by SET from the activated photocatalyst. The α-amino radical generated by reductive decarboxylation of a DMAT derivative with a redox-active ester (−1.26 V to −1.37 V vs a saturated calomel electrode) would enable turnover of the photoredox cycle (Figure 1b). Alternatively, we envisioned a more established approach expecting the direct oxidative photoredox decarboxylation of the carboxylic acid/carboxylate (by SET from the activated photocatalyst) of DMAT to generate the α-aminoalkyl radical that might readily be captured/trapped intramolecularly with the C4-pendant prenyl side-chain previously oxidized [76]. Closure of the photoredox catalytic cycle would then involve SET reduction, and protonation would deliver the desired carbocyclic ring (Figure 1c). If this cyclization reaction could be realized in either way, it would shorten the synthetic route of ergot alkaloids and may offer new opportunities for drug discovery and biochemistry applications.

**Scheme 1:**
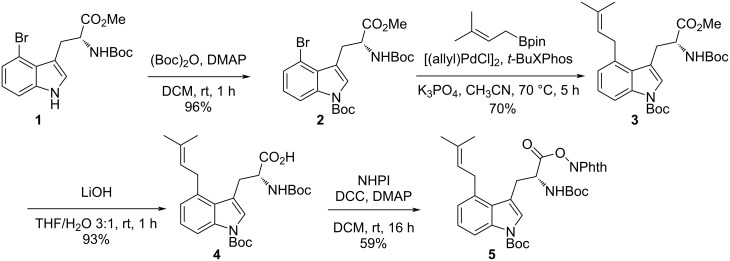
Synthesis of compound **5**.

As natural and unnatural tryptophan-derived redox-active *N*-hydroxyphthalimide esters have already been used in photoredox catalysis, we decided to use them as the initial substrates [77–85]. To test this concept, we turned our attention to the synthesis of key intermediate **5** (Scheme 1). The synthesis began with protection of the indole nitrogen of the known compound **1**, which is readily available from commercially available 4-bromoindole in one step [62]. Regioselective palladium-catalyzed prenylation of **2** with prenylboronic acid pinacol ester and subsequent hydrolysis with LiOH provided the linear prenylated acid **4** in good yield. Coupling acid **4** with *N*-hydroxyphthalimide using DCC and a catalytic amount of DMAP afforded the key intermediate **5** in 59% yield.

**Scheme 2:**
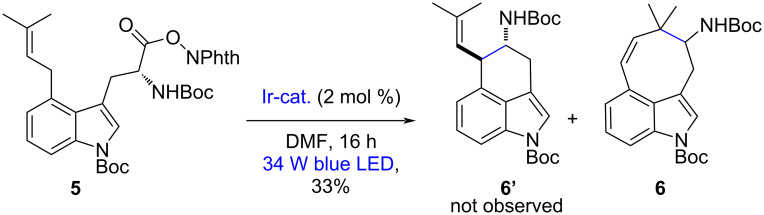
Photoredox-catalyzed radical decarboxylative cyclization of **5**.

**Figure 2:**
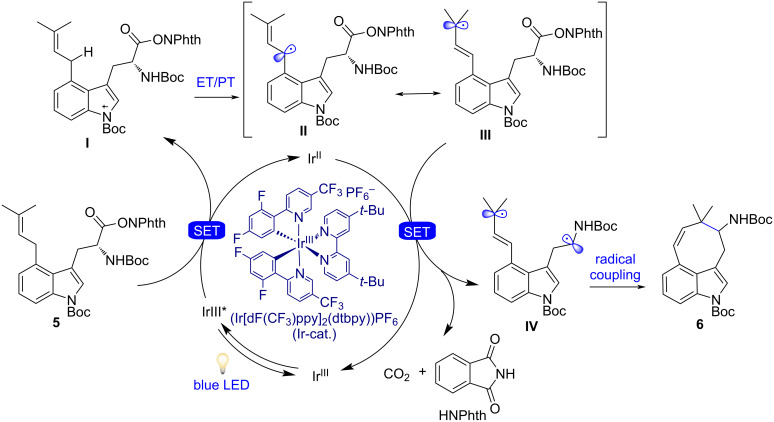
Proposed reaction mechanism for photoredox-catalyzed radical decarboxylative cyclization.

With compound **5** in hand, the required radical–radical coupling was investigated next, and some of the representative results are shown in Table S1 (see Supporting Information File 1). Irradiation from blue light-emitting diodes (LEDs) in the presence of 2 mol % of the photocatalyst [Ir(dF(CF_3_)ppy)_2_(dtbpy)]PF_6_ in CH_2_Cl_2_ at room temperature using our integrated photoreactor enabled efficient cyclization to give a decarboxylated compound with the correct mass (*m*/*z* 426.2) after 16 h. While we were delighted to find that the proposed radical–radical coupling in the synthesis of extracyclic fused indoles was feasible under these conditions, the observed reaction efficiency was poor (14–33% yield). However, on the ^1^H NMR spectrum, some unexpected signals were observed. The appearance of equilibrating species such as rotamers in the ^1^H NMR spectrum (see the variable-temperature NMR experiments in Supporting Information File 1, Figure S1) due to the protecting groups complicates the analysis of the reaction products. However, the olefinic signals were a pair of two doublets representing two vicinal vinylic protons [6.48 (d, *J* = 8.0 Hz, 1H), 6.29 (d, *J* = 8.0 Hz, 1H), 5.31 (d, *J* = 8.0 Hz, 1H), and 5.28 (d, *J* = 8.0 Hz, 1H)], strongly indicating that this product is not the desired structure **6’** but the eight-membered cycloalkene structure **6**, shown in Scheme 2. Based on these results and previous reports on the benzylic and allylic C–H bond functionalization enabled by metallaphotoredox catalysis [86], we propose a tentative mechanism (Figure 2).

First, the radical cation **I** was generated via the oxidation of indole **5** by the excited Ir-based photocatalyst, followed by sequential regioselective proton transfer on the benzylic dimethylallyl unit C–H bond of the C4 side-chain, thereby generating **II**. Here, the radical is stabilized by both the indole ring and the Δ2-olefin. Next, the resonance-stabilized radical intermediate **III** was trapped by the active α-aminoalkyl radical, generated by reductive decarboxylation by Ir(II) produced in the photocatalytic cycle (which undergoes oxidation to afford the Ir(III) complex and to close the cycle), thus furnishing compound **6** comprised of an eight-membered ring. The related stabilization effect of the conjugated product **6** might be the thermodynamic driving force for this radical coupling. An alternative route (not showen) would be that, the α-amidoalkyl radical generated by reductive decarboxylation, could add in an 8-*endo*-trig manner (common in radical chemistry) to the alkene and the resulting radical could be oxidized to the cation by the oxidized form of the photocatalyst to close the photocatalytic cycle, followed by formation of the double bond. Even though no desired cyclized product was observed, an interesting aspect of this reaction was the access of an attractive, unusual, and highly functionalized 3,4-fused eight-membered tricyclic indole, whose ring closure would not have been possible or at least very difficult in the ground state [87–89].

**Scheme 3:**
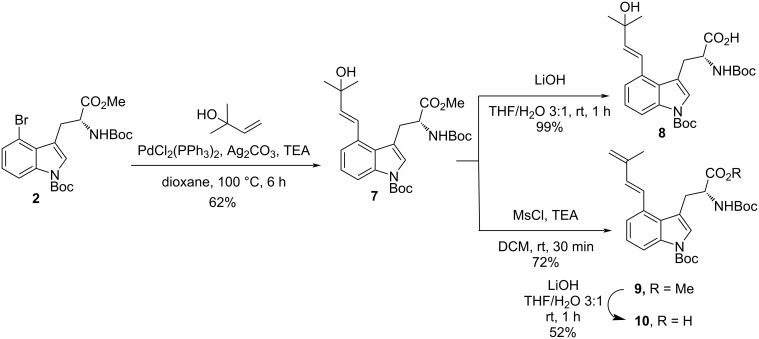
Synthesis of tryptophan derivatives **8** and **10**.

Although not yet completely clarified, some previous studies on the detailed mode of closure of the C ring in ergot alkaloids from DMAT have been shown to involve, before decarboxylative cyclization, oxidation on the C4-prenyl chain to give the stable rearranged allyl alcohol and/or the relatively unstable diene [90–91]. In addition, these results support the hypothesis that the decarboxylative cyclization can occur through subsequent selective 6-*exo*-trig radical addition. It also has been reported that it is difficult to detect which intermediate is really involved, since they are easily interconvertible to each other by hydration or dehydration, i.e., a plausible precursor of the allylic alcohol would be the diene, and vice versa [90]. Since both **8** and **10** are easily obtainable from **2** by Mozoroki–Heck coupling with commercially available 2-methyl-3-buten-2-ol, ester hydrolysis (LiOH in THF/H_2_O), and, finally for **10**, dehydration of the tertiary alcohol (mesylation and elimination) (Scheme 3), we decided to test their roles in the photoredox-catalyzed decarboxylative cyclization. With **8** and **10** in hand with the C4-prenyl side-chain already oxidized/functionalized, we recognized that this cyclization event would be triggered using their innate functionality, namely the α-amino carboxylate, through photoredox-mediated oxidative activation and CO_2_ extrusion, without the need for acid prefunctionalization to the redox-activated ester. Consequently, a technique involving direct generation of α-aminoalkyl radicals from free carboxylic acids of **8** and **10** under mild conditions would make the approach even more efficient and more biosimilar; nevertheless, issues regarding the regioselectivity of the ring formation could be raised, since both the 6-*exo*-trig and 7-*endo*-trig cyclization are both favorable, according to the Baldwin rules [92].

We began our investigation of the proposed decarboxylative cyclization by exposing the *N*-Boc derivative **8**, Ir catalyst, and K_2_HPO_4_ in DMF to a 34 W blue LED lamp at room temperature (Table 1) [93–97]. To our delight, cyclization was observed under these preliminary conditions, albeit in low yield (35% yield) and low regioselectivity (1:1) (Table 1, entry 1). No regiocontrol was observed; but remarkably, the regioisomers exhibited distinct retention factors on silica gel, allowing **11** and **12** to be isolated separately in good yield as single *trans* diastereomers [98]. Reducing the substrate concentration increased efficiency while assisting in avoiding the oligomerization pathways (Table 1, entries 2 and 3). Higher photocatalyst loadings resulted in an increased yield (Table 1, entry 4). Control experiments showed that both the photocatalyst and light were essential for product production (Table 1, entries 6 and 7), despite the fact that the removal of base did not result in a significantly reduced efficiency (Table 1, entry 5). The regioselectivity outcome was explained by the relative stability of the intermediate radicals involved, with strong evidence of the importance of steric effects [99]. Indeed, while the addition of the nucleophilic α-amino radical to the α-styrenyl position affords the 6-membered ring (kinetic product via intramolecular 6-*exo*-trig ring closure) [100] the resulting radical is unstabilized, the 7-membered ring (obtained via intramolecular 7-*endo*-trig ring closure) may well be the thermodynamic product based on the more stabilized benzylic radical that is produced [101].

**Table 1:** Photoredox-catalyzed radical decarboxylative cyclization of **8**.^a^

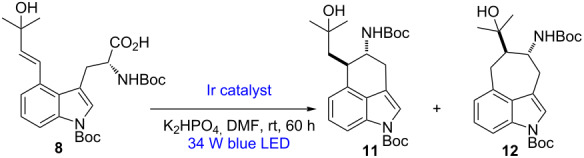					

Entry	Conditions	Concentration of **8**	Ir catalyst (mol %)	Ratio of **11**/**12**^b^	Yield of **11** and **12**^c^

1	as shown	10 mM	2	1:1	35%
2	as shown	5 mM	2	1:0.7	39%
3	as shown	2.5 mM	2	1:0.6	42%
4	as shown	2.5 mM	4	1:0.7	59%
5	no base	2.5 mM	4	1:0.7	53%
6	no photocatalyst	2.5 mM	–	–	N.D.^d^
7	no light	2.5 mM	4	–	N.D.^d^
8	DMSO instead of DMF	2.5 mM	4	0.7:1	33%
9	DCE instead of DMF	2.5 mM	4	1:0.7	40%

^a^Reaction conditions: **8** (0.1 mmol), K_2_HPO_4_ (0.12 mmol), catalyst (*x* mol %), solvent (4 mL), irradiation with 34 W blue LEDs for 60 h. ^b^Ratio of **11**/**12** was determined by ^1^H NMR analysis. ^c^Isolated yields. ^d^N.D., not detected.

As largely reported in the literature [102–103], radicals generated next to alcohols do not normally undergo β-elimination to give alkene/carbon–carbon double-bond formation and a hydroxyl radical (^•^OH). However, it is possible to transform an alcohol into a leaving group, in the radical sense, by converting it into a halide or pseudohalide derivative [104–105]. For alcohol **8**, all attempts to make a better leaving group, including phenyl sulfone derivative, to have radical addition–fragmentation on the latter and most likely to shift the regioselectivity towards 6-*exo*-trig by a favorable interplay of polar effects [99] failed and furnished only the 1,3-diene **10**. Unfortunately, when substrate **10** was subjected to the reaction conditions shown above, only tarry compounds were obtained; this result was probably due to the competitive cycloaddition and polymerization reactions and decomposition of the diene moiety, which is unstable and very sensitive to acidic and basic conditions [106].

**Figure 3:**
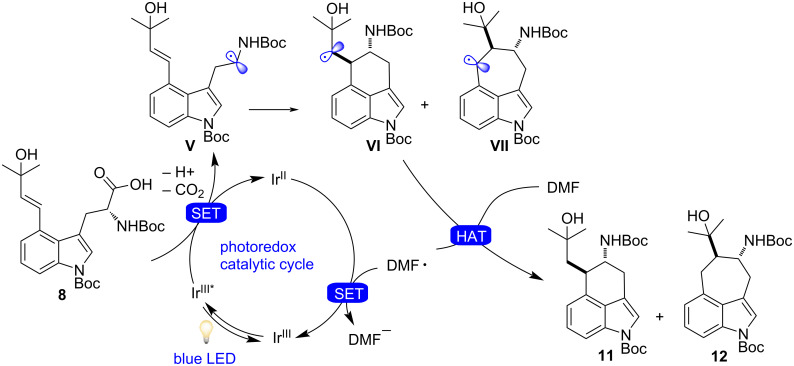
Proposed reaction mechanism for photoredox-catalyzed radical decarboxylative cyclization.

As shown in Figure 3 and anticipated above, our proposed mechanism begins with visible-light irradiation of the photoredox catalyst [Ir(dF(CF_3_)ppy)_2_(dtbpy)]PF_6_ to access the excited state *[Ir(dF(CF_3_)ppy)_2_(dtbpy)]PF_6_, which can trigger SET oxidation of **8**. Rapid decarboxylation leads to α-amino radical **V** (and the reduced photocatalyst), which is intercepted by the pendant double bond to forge the desired six-membered ring through a key C–C bond formation while furnishing secondary radical **VI** and the undesired seven-membered-ring compound **VII**. Closure of the photoredox catalytic cycle would then involve either SET reduction of the radical **VI** and **VII** (which upon protonation would deliver the desired product **11** and the undesired product **12**), or an hydrogen-atom-transfer (HAT) process (which would not place a formal negative charge onto the molecule), where the hydrogen atom required for this possible final HAT step originates from the solvent (DMF) itself [107]. Therefore, we tested the reaction in *N*,*N*-dimethylformamide-*d*_7_ (DMF-*d*_7_), which showed almost quantitative deuterium incorporation. While this result was surprising, further studies into this complex mechanism are ongoing and will be reported in due course.

**Scheme 4:**
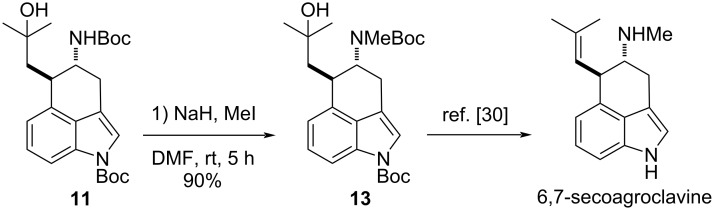
Methylation of **11** and the formal total synthesis of (±)-6,7-secoagroclavine.

The synthetic potential and utility of this method was further demonstrated by the formal total synthesis of (±)-6,7-secoagroclavine (Scheme 4) [108–114]. Towards this end, compound **11** was methylated efficiently and selectively at the secondary amide by treatment with methyl iodide in DMF to afford compound **13**. In additional two steps, intermediate **13** was transformed to (±)-6,7-secoagroclavine in enantiopure form, as reported previously by the Bisai group in 2018 [115]. All the spectroscopic data of **13** were in agreement with those reported in the literature, confirming that the radical addition reaction provided the *trans* amino group due to steric hindrance.

## Conclusion

In summary, this work illustrates, once more, the synthetic potential of an Ir-polypyridyl complex as a photoredox catalyst that can efficiently convert visible light into chemical energy. In addition, this catalyst was applied to demonstrate the proposed radical mechanism involved in the biosynthetic formation of the central C ring of several DMAT derivatives. The results presented here lend strong credence to decarboxylation and cyclization to form the six-membered ring as well as the nature of the stable oxidized intermediates concerned. Moreover, unprecedented and functionalized 3,4-fused tricyclic indoles with medium-sized rings (seven and eight), which have been largely neglected in previous studies, can be synthesized by this new protocol. Notably, the reaction has been successfully applied in the formal synthesis of (±)-6,7-secoagroclavine, a key intermediate for a common synthetic route to ergot alkaloids, providing an advantageous synthetic method for this class of natural products. Further studies on the utility of the decarboxylative radical cyclization and their applications are currently being investigated in our laboratory.

## Supporting Information

File 1 Experimental and copies of spectra.
