# Supplementary material for: Photoredox catalysis enabling decarboxylative radical cyclization of γ,γ-dimethylallyltryptophan (DMAT) derivatives: formal synthesis of 6,7-secoagroclavine
Source: Beilstein J Org Chem. 2023 Jun 26;19:918–27. doi: 10.3762/bjoc.19.70 (PMC10315889; doi:10.3762/bjoc.19.70)

BEILSTEIN JOURNAL OF ORGANIC CHEMISTRY

Supporting Information

for

Photoredox catalysis enabling decarboxylative radical cyclization of γ,γ-dimethylallyltryptophan (DMAT) derivatives: formal synthesis of 6,7-secoagroclavine

Alessio Regni, Francesca Bartoccini and Giovanni Piersanti

Beilstein J. Org. Chem. **2023,** *19,* 918–927. doi:10.3762/bjoc.19.70

Experimental and copies of spectra

License and Terms: This is a supporting information file under the terms of the Creative Commons Attribution License (https://creativecommons.org/licenses/by/4.0). Please note that the reuse, redistribution and reproduction in particular requires that the author(s) and source are credited and that individual graphics may be subject to special legal provisions.
The license is subject to the *Beilstein Journal of Organic Chemistry* terms and conditions: (https://www.beilstein-journals.org/bjoc/terms)

Table of contents

1. General methods S2
2. Starting material S2
3. Abbreviation S2
4. Table S1. Reaction optimization for photoredox-catalyzed radical decarboxylative cyclization of **5** S3
5. Synthesis and characterization data of compounds **2–13** S4
6. References S10
7. Copies of ^{1}H NMR and ^{13}C NMR spectra S11
8. Figure S1. Variable-temperature (VT) NMR experiments for compound **6** S23
9. Figure S2. MS spectra of **11** and **11-*d*** S24

General methods

All reactions were run in air unless otherwise noted. Column chromatography purifications were performed in flash chromatography conditions using 230–400 mesh silica gel. Analytical thin layer chromatography (TLC) was carried out on silica gel plates (Silica Gel 60 F254) that were visualized by exposure to ultraviolet light. The ^{1}H NMR and ^{13}C NMR spectra were recorded on a 400 spectrometer using $CDCl_3$, CD_3OD, DMSO-d_6 and acetone-d_6 as solvents. Chemical shifts (δ scale) are reported in parts per million (ppm) relative to the central peak of the solvent. Coupling constants (*J* values) are given in hertz (Hz). Structural assignments were made with additional information from the gCOSY experiment. Optical rotation analysis was performed with a polarimeter using a sodium lamp (λ = 589 nm, D-line); $[\alpha]_D^{20}$ values are reported in 10^{-1} deg cm^2 g^{-1}; concentration (c) is in g for 100 mL. HRMS analysis was performed using Orbitrap Exploris mass spectrometers.

Starting material

Methyl (*R*)-3-(4-bromo-1*H*-indol-3-yl)-2-((*tert*-butoxycarbonyl)amino)propanoate (**1**) was synthetized as reported in the literature [1].

Abbreviations

Boc_2O, di-*tert*-butyl dicarbonate; DCM, dichloromethane; DMAP, 4-(*N*,*N*-dimethylamino)pyridine; MsCl, methanesulfonyl chloride; TEA, triethylamine.

Table S1. Reaction optimization for photoredox-catalyzed radical decarboxylative cyclization of tryptophan-derived *N*-hydroxyphthalimide esters **5**.

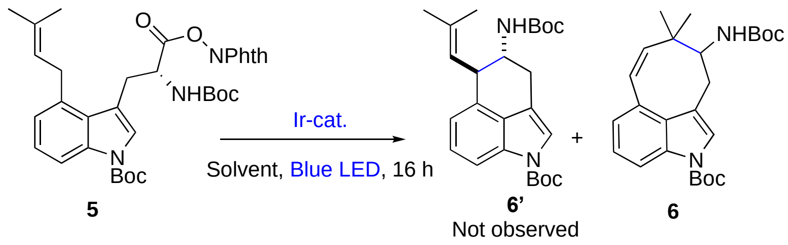

entry	solvent	concentration	Ir-cat (mol %)	yield
1	DCM	10 mM	2	14%
2	DCM	5 mM	2	21%
3	DCM	2.5 mM	2	33%
4	DMF	2.5 mM	2	N.D.
5	1,4-dioxane	2.5 mM	2	N.D.
6	DCM, with K_2HPO_4	2.5 mM	2	N.D.
7	DCM, no photocatalyst	2.5 mM	-	N.D.
8	DCM, no light	2.5 mM	2	N.D.

tert-Butyl (R)-4-bromo-3-(2-((tert-butoxycarbonyl)amino)-3-methoxy-3-oxopropyl)-1H-indole-1-carboxylate (2)

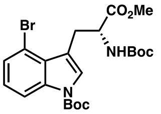

To a solution of (*R*)-3-(4-bromo-1*H*-indol-3-yl)-2-((*tert*-butoxycarbonyl)amino) propanoate **(+)-1** (1 g, 2.52 mmol) in dry DCM (34 mL) were added DMAP (15.4 mg, 0.126 mmol) and Boc_2O (604 mg, 2.77 mmol). The reaction mixture was stirred at room temperature for 1 h and then diluted with DCM (34 mL) and an aqueous solution of HCl 0.1 N (34 mL). The organic phase was separated, and the aqueous phase was extracted with DCM (2 × 15 mL). The combined organic phases were washed with brine, dried over anhydrous Na_2SO_4, and concentrated under reduced pressure. The residue obtained was purified by flash chromatography (cyclohexane/EtOAc 8 : 2) to obtain **2** (1.2 g, 96%) as a white solid.

^{1}H NMR (400 MHz, $CDCl_3$): δ 8.16 (d, *J* = 8.0 Hz, 1H), 7.48 (s, 1H), 7.38 (d, *J* = 8.0 Hz, 1H), 7.12 (t, *J* = 8 Hz, 1H), 5.15 (br d, *J* = 8.5 Hz,1H), 4.71 (m, 1H), 3.74 (s, 3H), 3.64 (dd, *J* = 15.0, 5.0 Hz, 1H), 3.23 (dd, *J* = 15.0, 9.0 Hz, 1H),1.65 (s, 9H), 1.36 (s, 9H);

The chemical−physical data are in accordance with the literature [2].

tert-Butyl (R)-3-(2-((tert-butoxycarbonyl)amino)-3-methoxy-3-oxopropyl)-4-(3-methylbut-2-en-1-yl)-1H-indole-1-carboxylate (3)

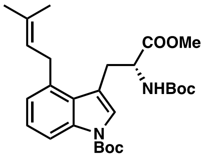

To a solution of compound **2** (100 mg, 0.2 mmol) in CH_3CN (0.4 mL) was added [(allyl)PdCl]$_2$ (2.2 mg, 0.006 mmol), 2-di-*tert*-butylphosphino-2',4',6'-triisopropylbiphenyl (10 mg, 0.024 mmol), K_3PO_4 (2.5 M, 0.4 mL) and 3-methyl-2-butenylboronic acid pinacol ester (46 mg, 0.24 mmol). The reaction mixture was evacuated and backfilled with argon for three times, and then stirred at 70 °C for 5 h. After cooling to room temperature, the organic phase was separated. The aqueous phase was extracted with EtOAc (2 x 5 mL). The combined organic layer was washed with brine (10 mL), dried over anhydrous Na_2SO_4, and concentrated under reduced pressure. The crude product was purified by flash chromatography (cyclohexane/EtOAc 8 : 2) to provide compound **3** (68 mg, 70%) as yellowish solid.

^{1}H NMR (400 MHz, $CDCl_3$): δ 8.07 (d, *J* = 8.5 Hz, 1H), 7.42 (s, 1H), 7.23 (t, *J* = 8.5 Hz, 1H), 7.12 – 6.94 (m, 1H), 5.27 (br s, 1H), 5.05 (d, *J* = 8.5 Hz, 1H), 4.68 4.64 (m, 1H), 3.74 (s, 3H), 3.70 (t, *J* = 8.0 Hz, 2H), 3.42 (dd, *J* = 15.5, 4.5 Hz, 1H), 3.17 (dd, *J* = 15.5, 8.5 Hz, 1H), 1.75 (d, J = 2.9 Hz, 10H), 1.76 (s, 3H), 1.75 (s,3H), 1.65 (s, 9H), 1.42 (s, 9H).

^{13}C{H} NMR (100 MHz, $CDCl_3$): δ 172.8, 155.2, 149.4, 136.2, 134.7, 132.7, 128.0, 124.5, 124.2, 123.6, 123.4, 115.7, 113.3, 83.5, 80.0, 54.0, 52.3, 32.0, 30.1, 28.3, 28.2, 25.6, 18.0

$[\alpha]_D^{25}$= -11.1 (c = 0.316, $CHCl_3$)

HRMS (ESI-TOF) *m*/*z*: $[M + Na]^+$ calcd for $C_{27}H_{38}N_2NaO_6$, 509.2622; found, 509.2625.

(*R*)-3-(1-(*tert*-Butoxycarbonyl)-4-(3-methylbut-2-en-1-yl)-1*H*-indol-3-yl)-2-((*tert*-butoxycarbonyl)amino)propanoic acid (4)

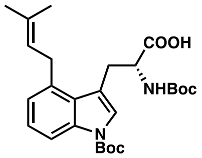

To a solution of **3** (74 mg, 0.15 mmol) in THF/H_2O 3:1 (2.2 mL) was added LiOH·H_2O (19.2 mg, 0.46 mmol). The mixture was stirred at room temperature for 1 h. The reaction mixture was acidified with an aqueous solution of $KHSO_4$ 3 M (pH 2) and diluted with EtOAc. The two phases were separated, and the aqueous phase was extracted with EtOAc (2 × 10 mL), the combined organic phases were washed with brine, dry over Na_2SO_4 and the solvents evaporated under reduced pressure. The crude product was purified by flash chromatography (DCM/MeOH 95:15) to obtain **4** (66 mg, 93%) as a white solid.

^{1}H NMR (400 MHz, CD_3OD): δ 8.02 (d, *J* = 8.0 Hz, 1H), 7.46 (s, 1H), 7.16 (t, *J* = 8.0 Hz, 1H), 6.97 (d, *J* = 8.0 Hz, 1H), 5.28-5.25 (m, 1H), 4.43 – 4.43 (m, 1H), 3.74 -3.73 (m, 2H), 3.47 (d, *J* = 14.0 Hz, 1H), 3.04 (dd, *J* = 15.0, 9.5 Hz, 1H), 1.74 (s, 6H), 1.65 (s, 9H), 1.37 (s, 9H);

^{13}C{H} NMR (100 MHz, $CDCl_3$): δ 175.9, 155.8, 149.5, 136.2, 134.8, 132.9, 128.0, 124.5, 124.3, 123.6, 123.3, 115.8, 113.3, 83.6, 80.5, 54.0, 32.0, 29.6, 28.2, 27.5, 25.7, 18.0;

$[\alpha]_D^{25}$= +19.8 (c = 0.418, MeOH)

HRMS (ESI-TOF) *m*/*z*: $[M + Na]^+$ calcd for $C_{26}H_{36}N_2NaO_6$, 495.2466; found, 495.2470.

tert-Butyl (*R*)-3-(2-((*tert*-butoxycarbonyl)amino)-3-((1,3-dioxoisoindolin-2-yl)oxy)-3-oxopropyl)-4-(3-methylbut-2-en-1-yl)-1*H*-indole-1-carboxylate (5)

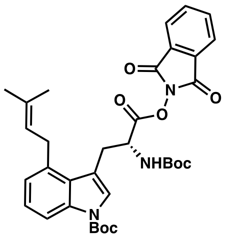

In a well-dried reaction flash, equipped with a stirring bar under N_2, was added **4** (65 mg, 0.138 mmol) in a solution of dry DCM (0.4 mL, 0.15 M), followed by DMAP (0.84 mg, 0.007 mmol) and *N*-hydroxyphthalimide (24.6 mg, 0.151 mmol). The mixture was stirred until complete dissolution of the solids and afterwards was added dropwise a solution of DCC (31.16 mg, 0.151 mmol) in dry DCM (0.2 mL). The reaction was stirred at room temperature for 16 h. The crude of the reaction was filtered, and the resulting solution was evaporated under reduced pressure. The crude product was purified by flash chromatography (DCM/EtOAc 95 : 5) to obtain **5** (50 mg, 59%) as a yellowish solid.

^{1}H NMR (400 MHz, $CDCl_3$): δ 8.12 (d, *J* = 8.5 Hz, 1H), 7.89 – 7.88 (m, 2H), 7.82 – 7.80 (m, 2H), 7.67 (s, 1H), 7.24 (d, *J* = 7.5 Hz, 1H), 7.06 (d, *J* = 7.5 Hz, 1H), 5.30 (s, 1H), 5.08 (s, 1H), 3.80 (dd, *J* = 16.5, 6.5 Hz, 1H), 3.71 (d, *J* = 14.5 Hz, 2H), 3.39 – 3.34 (m, 1H), 1.77 (s, 3H), 1.75 (s, 3H), 1.68 (s, 9H), 1.42 (s, 9H).

^{13}C{H} NMR (100 MHz, $CDCl_3$): δ 168.9, 161.4, 154.8, 149.4, 136.4, 134.8, 134.6, 133.1, 128.9, 127.9, 125.3, 124.6, 124.0, 123.7, 123.4, 114.3, 113.3, 83.5, 80.6, 52.6, 32.1, 29.9, 28.2, 26.9, 25.6, 18.1.

$[\alpha]_D^{25}$= + 24.3 (c = 0.415, $CHCl_3$)

HRMS (ESI-TOF) *m*/*z*: [M + Na]$^+$ calcd for $C_{34}H_{39}N_3NaO_8$, 640.2629; found, 640.2676.

tert-Butyl (*R*,*Z*)-9-((*tert*-butoxycarbonyl)amino)-8,8-dimethyl-9,10-dihydrocycloocta[*cd*]indole-2(8*H*)-carboxylate (6)

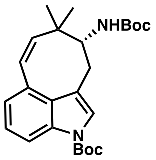

In a well-dried hermetic vial, equipped with a stirring bar, was added *tert*-butyl (*R*)-3-(2-((*tert*-butoxycarbonyl)amino)-3-((1,3-dioxoisoindolin-2-yl)oxy)-3-oxopropyl)-4-(3-methylbut-2-en-1-yl)-1*H*-indole-1-carboxylate (**5**) (35 mg, 0.081 mmol), followed by Ir[dF(CF_3)ppy]$_2$(dtbpy)PF_6 (1.83 mg, 0.0016 mmol). The vial was hermetically close and degassed with repetition of 3vacuum/N_2 cycles. Degassed DMF (3.3 mL, 0.025M) was syringed in the vial and the mixture was irradiated with blue led for 16 h. The crude was diluted with EtOAc and H_2O, the two phases was separated, and the organic phase was washed with H_2O (2 x 10 mL), dry over Na_2SO_4 and the solvent evaporated under reduced pressure. The crude product was purified by flash chromatography (cyclohexane/EtOAc 8:2) to obtain **6** (12 mg, 33%) as a yellowish solid.

^{1}H NMR (400 MHz, $CDCl_3$). (mixture of rotamers) δ 8.09 (d, *J* = 8.0 Hz, 1H), 8.04 (d, *J* = 8.0 Hz, 1H), 7.38 (s, 2H), 7.25 –7.21 (m, 2H), 6.99 (d, *J* = 7.5 Hz, 1H), 6.93 (d, *J* = 7.5 Hz, 1H), 6.48 (d, *J* = 8.0 Hz, 1H), 6.29 (d, *J* = 8.0 Hz, 1H), 5.31 (d, *J* = 8.0 Hz, 1H), 5.28 (d, *J* = 8.0 Hz, 1H), 4.07 – 4.00 (m, 1H), 3.90 – 3.86 (m, 1H), 3.48 – 3.36 (m, 2H), 3.25 -3.11 (m, 2H), 1.87 (s, 6H), 1.73 (s, 6H), 1.66 (s, 18H), 1.47 (s, 9H), 1.45 (s, 9H);

^{13}C{H} NMR (100 MHz, $CDCl_3$) 154.7, 154.4, 149.5, 137.9, 137. 7, 136.5, 136.4, 135.5, 127.2, 127.23, 125.3, 125.2, 124.1, 124.0, 122.7, 122.6, 121. 8, 121.2, 119.3, 119.0, 113.3, 113.1, 83.3, 79.8, 79.5, 58.1, 57.0, 42.2, 41.9, 29.70, 28.5, 28.4, 28.2, 27.7, 26.9, 25.7;

HRMS (ESI-TOF) *m*/*z*: [M + Na]$^+$ calcd for $C_{25}H_{34}N_2NaO_4$, 449.2411; found, 449.2417.

***tert*-Butyl (*R*,*E*)-3-(2-((*tert*-butoxycarbonyl)amino)-3-methoxy-3-oxopropyl)-4-(3-hydroxy-3-methylbut-1-en-1-yl)-1*H*-indole-1-carboxylate (7)**

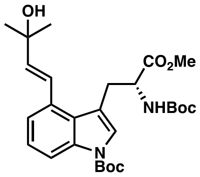

To a solution of **2** (305 mg, 0.61 mmol) in 1,4-dioxane (3 mL) were added 2-methyl-3-buten-2-ol (0.37 mL, 3.56 mmol), TEA (0.73 mL, 4.91 mol), Ag_2CO_3 (186 mg, 0.67 mmol), and $PdCl_2(PPh_3)_2$ (43 mg, 0.061 mmol) in succession. The reaction mixture was evacuated and backfilled with argon three times and then stirred at 100 °C for 6 h. After cooling to room temperature, the reaction mixture was poured into H_2O (5 mL). The organic layer was separated, and the aqueous layer was extracted with EtOAc (2 × 10 mL). The combined organic layer was washed with brine (20 mL), dried over anhydrous Na_2SO_4, and concentrated under reduced pressure. The residue was purified by flash chromatography (cyclohexane/EtOAc 7 : 3) to obtain **7** (188 mg, 62%) as a white solid.

^{1}H NMR (400 MHz, $CDCl_3$) δ 8.09 (s, 1H), 7.35−7.31 (m, 2H), 7.28−7.24 (m, 2H), 6.33 (d, *J* = 15.5 Hz, 1H), 5.17 (br d, *J* = 8.5 Hz, 1H), 4.61 (m, 1H), 3.57 (s, 3H), 3.30 (dd, *J* = 14.0, 7.0 Hz, 1H), 3.16 (dd, *J* = 14.0, 8.0 Hz, 1H), 1.66 (s, 9H), 1.46 (s, 3H), 1.45 (s, 3H), 1.41 (s, 9H);

The chemical−physical data are in accordance with the compound reported in the literature [3].

(*R*,*E*)-3-(1-(*tert*-Butoxycarbonyl)-4-(3-hydroxy-3-methylbut-1-en-1-yl)-1*H*-indol-3-yl)-2-((*tert*-butoxycarbonyl)amino)propanoic acid (8)

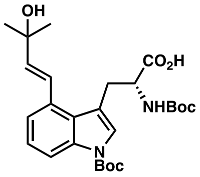

To a solution of **7** (500 mg, 1 mmol) in THF/H_2O 3:1 (14.5 mL) was added LiOH·H_2O (126 mg, 3 mmol). The mixture was stirred at room temperature for 1 h. The reaction mixture was acidified with an aqueous solution of $KHSO_4$ 3 M (pH 2) and diluted with EtOAc. The two phases were separated, and the aqueous phase was extracted with EtOAc (2 × 40 mL). The combined organic phases were washed with brine, dry over Na_2SO_4 and the solvent evaporated under reduced pressure. The residue obtained was purified by flash chromatography (DCM/MeOH 9:1) to obtain **8** (480 mg, 99%) as a white solid.

^{1}H NMR (400 MHz, DMSO-*d*$_6$) δ 8.02 (t, *J* = 4.0 Hz, 1H), 7.51 (s, 1H), 7.32 – 7.28 (m, 3H), 6.79 (br s, 1H), 6.33 (d, *J* = 16.0 Hz, 1H), 4.19 – 4.13 (m, 1H), 3.40 (dd, *J* = 15.0, 4.0 Hz, 1H), 2.93 (dd, *J* = 15.0, 11.0 Hz, 1H), 1.65 (s, 9H), 1.37 (s, 3H), 1.36 (s, 3H), 1.33 (s, 9H).

^{13}C{H} NMR (100 MHz, DMSO-*d*$_6$) δ 174.8, 155.6, 149.4, 142.8, 136.1, 132.4, 127.8, 125.1, 124.7, 123.3, 120.8, 118.4, 113.8, 83.8, 78.0, 70.0, 55.1, 30.5, 30.2, 28.6, 28.2, 27.6;

$[\alpha]_D^{25}$= -17.3 (c = 0.434, MeOH);

HRMS (ESI-TOF) *m*/*z*: $[M + Na]^+$ calcd for $C_{26}H_{36}N_2NaO_7$, 511.2415; found, 511.2423.

***tert*-Butyl (*R,E*)-3-(2-((*tert*-butoxycarbonyl)amino)-3-methoxy-3-oxopropyl)-4-(3-methylbuta-1,3-dien-1-yl)-1*H*-indole-1-carboxylate (9)**

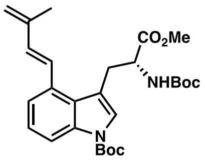

To a solution of **7** (100 mg, 0.2 mmol) in DCM (2 mL) were added TEA (111 µL, 0.8 mmol) and MsCl (62 µl, 0.8 mmol). The reaction mixture was stirred at room temperature for 30 minutes. The reaction mixture was diluted with DCM (10 mL) and H_2O (10 mL). The organic layer was separated, and the aqueous layer was extracted with DCM (2 × 10 mL). The combined organic layer was washed with brine (20 mL), dried over anhydrous Na_2SO_4, and concentrated under reduced pressure. The residue was purified by flash chromatography (cyclohexane/EtOAc 8 : 2) to obtain **9** (70 mg, 72%) as a white solid.

^{1}H NMR (400 MHz, $CDCl_3$) δ 8.11 (d, *J* = 8.0 Hz, 1H), 7.43 (s, 1H), 7.36 (d, *J* = 7.5 Hz, 1H), 7.28 (t, *J* = 7.5 Hz, 1H), 7.20 (d, *J* = 16.0 Hz, 1H), 6.82 (d, *J* = 16.0 Hz, 1H), 5.13 (d, *J* = 15.0 Hz, 2H), 5.05 (br d, *J* = 8.0 Hz, 1H), 4.64 – 4.58 (m,1 H), 3.73 (s, 3H), 3.46 (dd, *J* = 15.0, 5.0 Hz, 1H), 3.17 (dd, *J* = 15.0, 8.5 Hz, 1H), 2.05 (s, 3H), 1.67 (s, 9H), 1.37 (s, 9H).

^{13}C{H} NMR (100 MHz, $CDCl_3$) δ 172.7, 155.1, 149.3, 142.2, 136.3, 134.8, 131.9, 127.3, 126.1, 125.1, 124.6, 120.9, 117.6, 115.7, 114.4, 83.7, 79.9, 77.2, 53.8, 52.2, 30.6, 28.2, 18.5.

$[\alpha]_D^{25}$= - 10.1 (c = 0.119, $CHCl_3$)

HRMS (ESI-TOF) *m/z*: $[M + Na]^+$ calcd for $C_{27}H_{36}N_2NaO_6$, 507.2466; found, 507.2469.

(*R*,*E*)-3-(1-(*tert*-Butoxycarbonyl)-4-(3-methylbuta-1,3-dien-1-yl)-1*H*-indol-3-yl)-2-((*tert*-butoxycarbonyl)amino)propanoic acid (10)

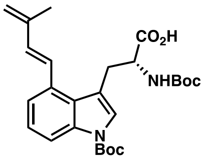

To a solution of **9** (210 mg, 0.43 mmol) in THF/H_2O 3:1 (6.2 mL) was added LiOH·H_2O (54.7 mg, 1.3 mmol). The mixture was stirred at room temperature for 1 h. The reaction mixture was acidified with an aqueous solution of $KHSO_4$ 3 M (pH 2) and diluted with EtOAc. The two phases were separated, and the aqueous phase was extracted with EtOAc (2 × 40 mL). The combined organic phases were washed with brine, dry over Na_2SO_4 and the solvent evaporated under reduced pressure. The residue obtained was purified by flash chromatography (DCM/MeOH 9:1) to obtain **10** (105 mg, 52%) as a yellowish solid.

^{1}H NMR (400 MHz, $CDCl_3$) δ 8.11 (d, *J* = 8.0 Hz, 1H), 7.48 (s, 1H), 7.37 (d, *J* = 8.0 Hz, 1H), 7.29 (t, *J* = 8.0 Hz, 1H), 7.19 (d, *J* = 16.0 Hz, 1H), 6.83 (m, *J* = 16.0 Hz, 1H), 5.14 (d, *J* = 17.0 Hz, 2H), 5.03

(br d, J = 7.0 Hz, 1H), 4.57 (br s, 1H), 3.65 – 3.56 (m, 1H), 3.23 –3.17 (m, 1H), 2.04 (s, 3H), 1.62 (s, 9H), 1.38 (s, 9H).

^{13}C{H} NMR (100 MHz, $CDCl_3$) δ 175.7, 155.8, 149.3, 142.2, 136.4, 135.1, 132.1, 127.3, 125.9, 125.3, 124.7, 121.1, 117.9, 115.6, 114.5, 83.8, 80.5, 54.4, 53.8, 28.1, 27.4, 18.5.

$[\alpha]_D^{25}$= + 16.4 (c = 0.085, $CHCl_3$)

HRMS (ESI-TOF) *m*/*z*: $[M + Na]^+$ calcd for $C_{26}H_{34}N_2NaO_6$, 493.2309; found, 493.2312.

***tert*-Butyl (4*R*,5*R*)-4-((*tert*-butoxycarbonyl)amino)-5-(2-hydroxy-2-methylpropyl)-4,5-dihydrobenzo[*cd*]indole-1(3*H*)-carboxylate (11) and *tert*-butyl (7*R*,8*R*)-8-((*tert*-butoxycarbonyl)amino)-7-(2-hydroxypropan-2-yl)-6,7,8,9-tetrahydro-2*H*-cyclohepta[*cd*]indole-2-carboxylate (12)**

In a manner analogous to the procedure reported by MacMillan [4], a well-dried hermetic vial, equipped with a stirring bar, was added **8** (150 mg, 0.31 mmol) followed by $Ir[dF(CF_3)ppy]_2(dtbpy)PF_6$ (14 mg, 0.0124 mmol) and K_2HPO_4 (65 mg, 0.37 mmol). The vial was hermetically close and degassed with vacuum/N_2 cycles (3 times). Degassed DMF (12.4 mL) was syringed in the vial and the mixture was irradiated with blue led for 60 h. The crude was diluted with EtOAc and H_2O, the two phases were separated, and the organic phase was washed with H_2O, dry over Na_2SO_4 and the solvent evaporated under reduced pressure. The crude product was purified by flash-chromatography (gradient from DCM/EtOAc 9:1 to DCM/EtOAc 7:3) to afford **11** and **12** as mixture of separable regioisomers (ratio **11**:**12** 1:0.7). **11** (48 mg, 35%) and **12** (33 mg, 24%) as a yellowish solid.

11

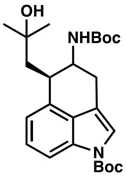

^{1}H NMR (400 MHz, Acetone-d_6) δ 7.64 (br d, J = 8.0 Hz, 1H), 7.22 (s, 1H), 7.13 (t, J = 7.5 Hz, 1H), 6.96 (d, J = 7.5 Hz, 1H), 5.41 (br d, J = 7.5 Hz, 1H), 4.34 4.31 (m, 1H), 3.48 (s, 1H), 3.22 3.18 (m, 1H), 3.21-3.16 (m, 1H), 3.08 – 3.03 (m, 1H), 2.73 –2.67 (m, 2h), 2.70 (s, 3H), 1.67 (dd, J = 15.0, 8.0 Hz, 1H), 1.55 (dd, J = 15.0, 3.5 Hz, 1H), 1.54 (s, 9H), 1.28 (s, 3H), 1.20 (s, 9H), 1.18 (s, 3H);

^{13}C{H} NMR (100 MHz, Acetone-d_6) δ 155.3, 149.7, 135.0, 133.5, 128.0, 125.2, 120.8, 120.4, 114.7, 112.6, 82.9, 77.7, 69.9, 50.1, 48.1, 39.0, 30.7, 28.4, 27.7, 27.4, 23.8;

HRMS (ESI-TOF) *m*/*z*: $[M + Na]^+$ calcd for $C_{25}H_{36}N_2NaO_5$, 467.2516; found, 467.2523.

12

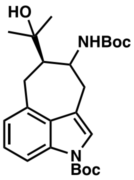

^{1}H NMR (400 MHz, Acetone-d_6) δ 7.88 (d, J = 8.0 Hz, 1H), 7.34 (s, 1H), 7.08 (t, J = 8.0 Hz), 6.96 (d, J = 8.0 Hz, 1H), 5.45 (br d, J = 9.0 Hz, 1H), 4.56 – 4.51 (m, 1H), 3.63 (s, 1H), 3.21 –3.16 (m, 1H), 3.07 – 3.00 (m, 2H), 2.86 – 2.80 (m, 1H), δ = 1.89 - 188 (m, 1H), 1.53 (s, 9H), 1.22 (s, 9H), 1.17 (s, 6H);
$^{13}C\{H\}$ NMR (100 MHz, $CDCl_3$) δ 155.8, 149.2, 136.3, 135.0, 128.9, 124.0, 123.7, 123.0, 116.8, 112.3, 83.1, 78.2, 71.8, 53.5, 47.8, 34.3, 30.8, 27.7, 27.4, 27.2, 26.9;

HRMS (ESI-TOF) *m/z*: $[M + Na]^+$ calcd for $C_{25}H_{36}N_2NaO_5$, 467.2516; found, 467.2547.

***tert*-Butyl (4*R*,5*R*)-4-((*tert*-butoxycarbonyl)(methyl)amino)-5-(2-hydroxy-2-methylpropyl)-4,5-dihydrobenzo[*cd*]indole-1(3*H*)-carboxylate (13)**

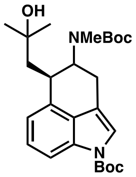

To a solution of **11** (38 mg, 0.086 mmol) in DMF dry (0.45 mL) under N_2, was added NaH (6.16 mg, 0.257 mmol, 60% in mineral oil). The mixture was stirred for 5 min at room temperature and after culled at 0 °C, was added dropwise MeI (27 μL, 0.143 mmol) The reaction mixture was stirred at room temperature for 5 h. The reaction was quenched with H_2O and EtOAc, the two phases were separated, and the aqueous phase was extracted with EtOAc (2 × 10 mL), dry over Na_2SO_4 and the solvent evaporated under reduced pressure. The crude product was purified by flash-chromatography (cyclohexane/EtOAc 8:2) to obtain **13** (35 mg, 90%) as a yellowish solid.

^{1}H NMR (400 MHz, Acetone-d_6) δ 7.78 (br s, 1H), 7.34–7.27 (m, 3H), 4.59–4.53 (m, 1H), 3.34–3.29 (m, 1H), 3.09–3.05 (m, 2H), 2.68 (s, 3H), 1.93 (dd, *J* = 14.5, 4.5 Hz 1H), 1.85–1.82 (m, 1H), 1.68 (s, 9H), 1.46 (s, 9H), 1.39 (s, 3H), 1.30 (s, 3H);
$^{13}C\{H\}$ NMR (100 MHz, Acetone-d_6) δ 149.8, 136.7, 133.1, 128.3, 125.3, 120.7, 118.7, 116.4, 112.3, 83.0, 78.9, 69.6, 69.5, 55.9, 46.8, 37.1, 30.2, 27.7, 27.4, 26.6;
HRMS (ESI-TOF) m/z: $[M + Na]^+$ calcd for $C_{26}H_{38}N_2NaO_5$, 481.2673; found, 481.2675.

References

1. Bartoccini, F.; Fanini, F.; Retini, M.; Piersanti, G. *Tetrahedron Lett.* **2020**, *61*, 151923.
2. Tao, S.; Caiyun, M.; Guangyan, Z.; Xuan, P.; Zhanzhu, L. *J. Nat. Prod.* **2022**, *85*, 1128-1133.
3. Ma, Y.; Yan, J.; Yang, L.; Yao, Y.; Wang, L.; Gao, S.-S.; Cui, C. *Front. Bioeng. Biotechnol.* **2022**, *10*:1095464.
4. McCarver, S. J.; Qiao, J. X.; Carpenter, J.; Borzilleri, R. M.; Poss, M. A.; Eastgate, M. D.; Miller, M. M.; MacMillan, D. W. C. *Angew. Chem., Int. Ed.* **2017**, *56*, 728–732.

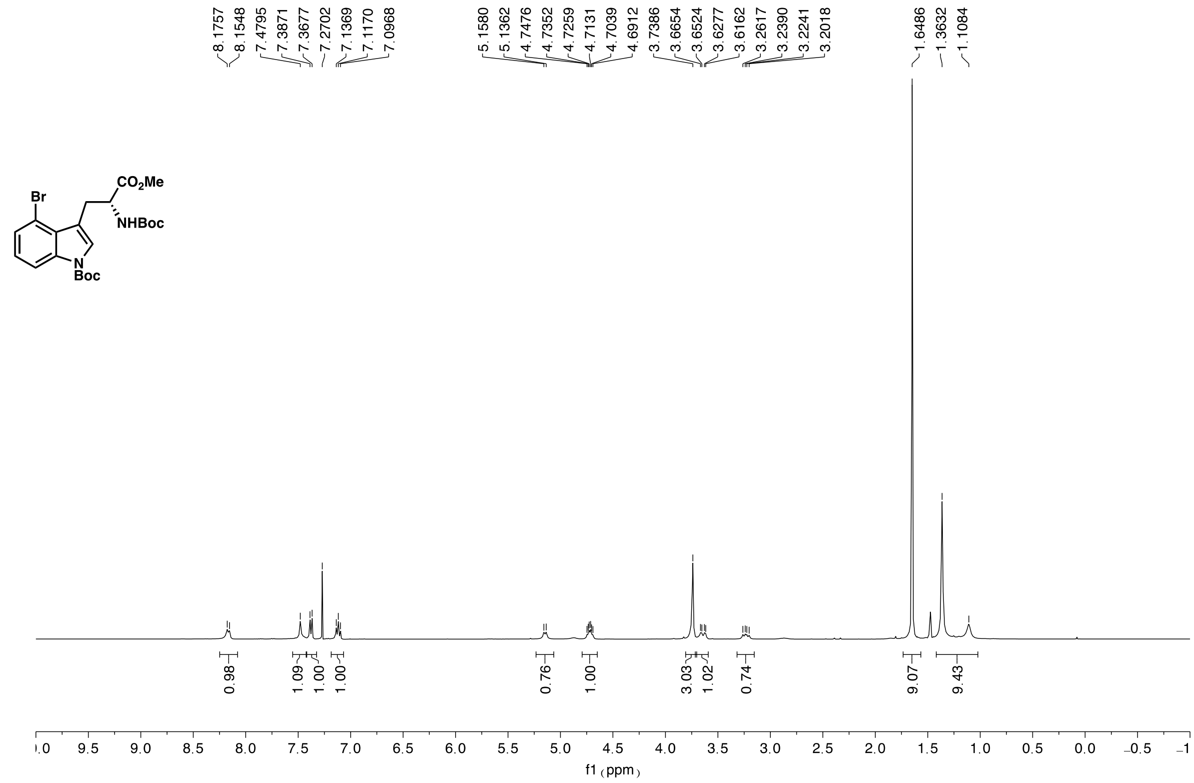

^{1}H NMR spectrum (400 MHz, $CDCl_3$) of compound 2

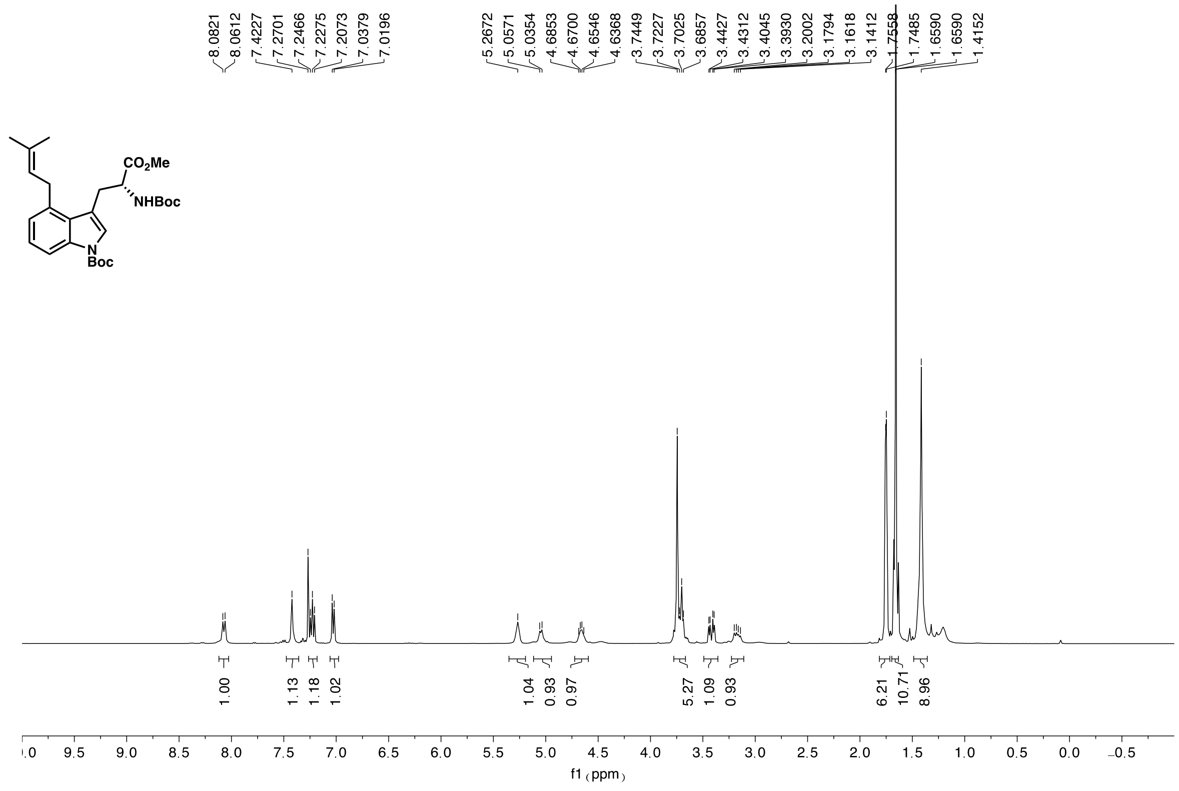

^{1}H NMR spectrum (400 MHz, $CDCl_3$) of compound 3

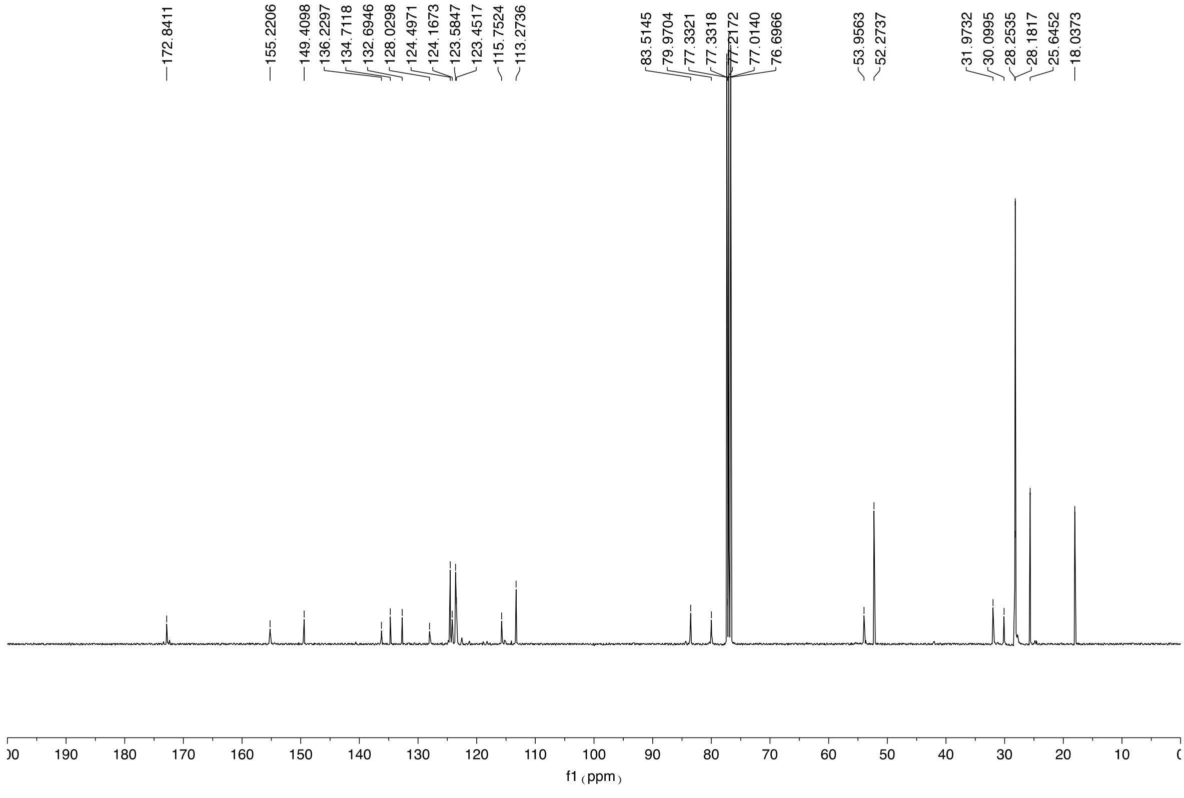

^{13}C{H} NMR spectrum (100 MHz, $CDCl_3$) of compound 3

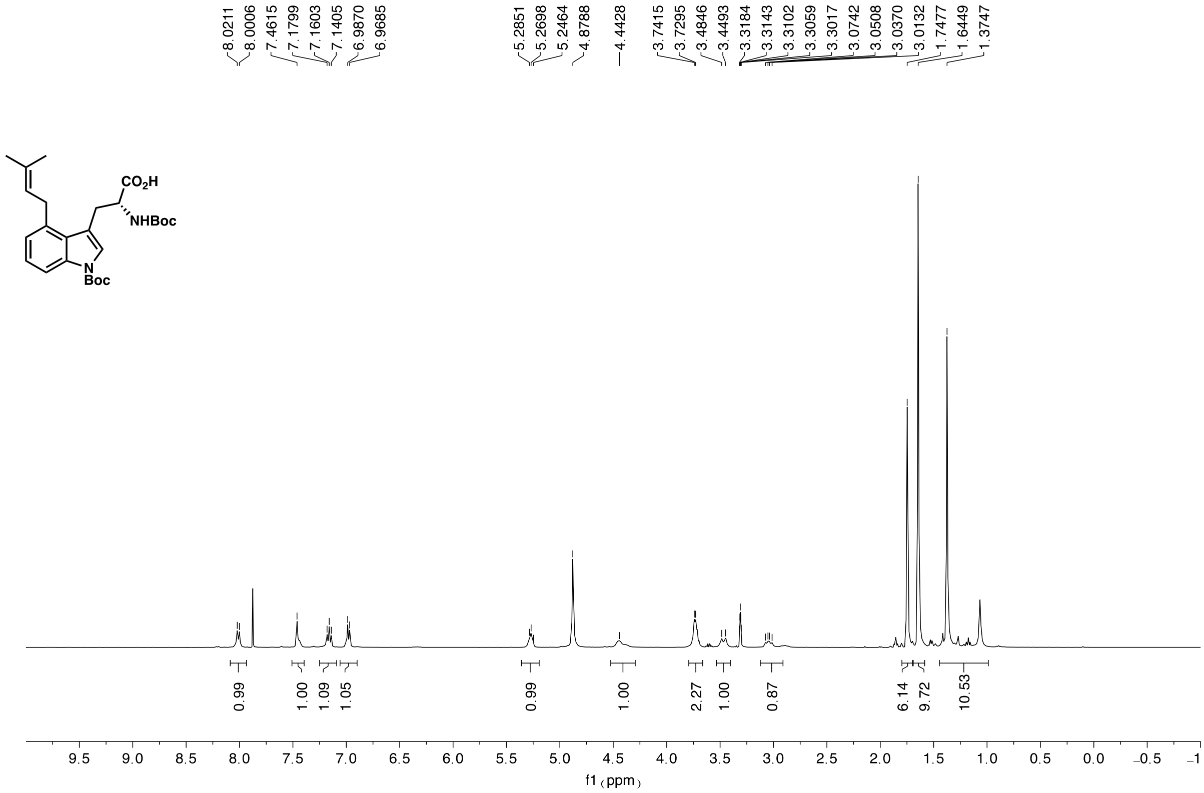

^{1}H NMR spectrum (400 MHz, CD_3OD) of compound 4

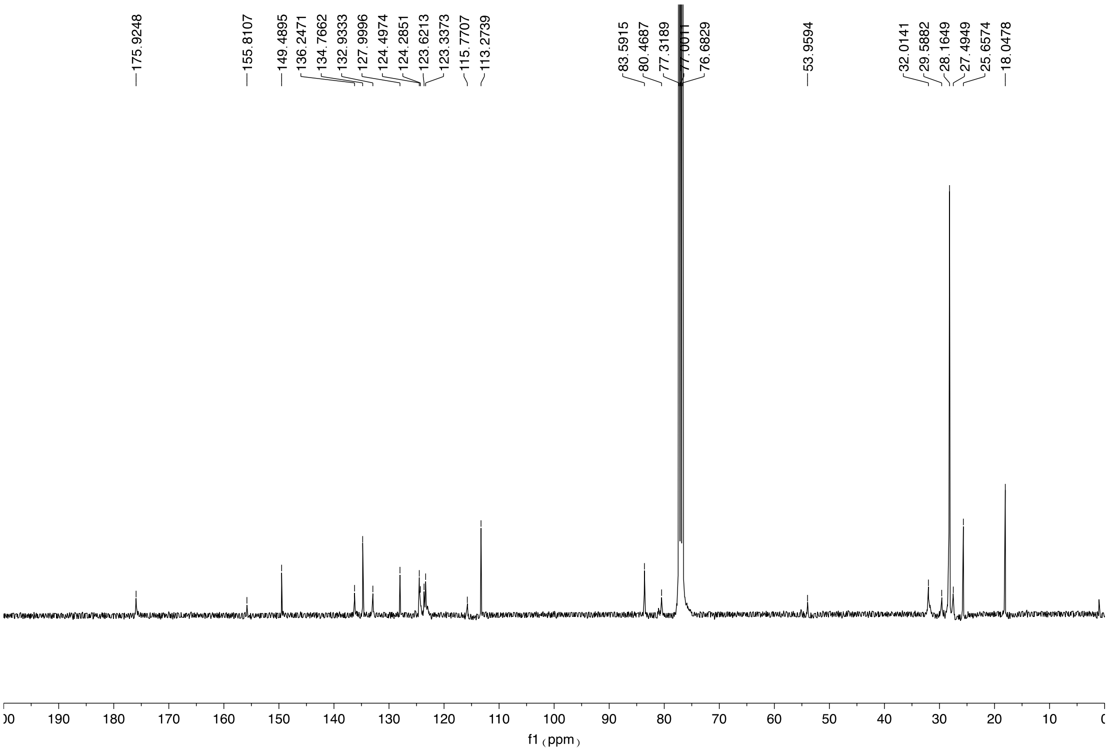

$^{13}C\{H\}$ NMR spectrum (100 MHz, $CDCl_3$) of compound 4

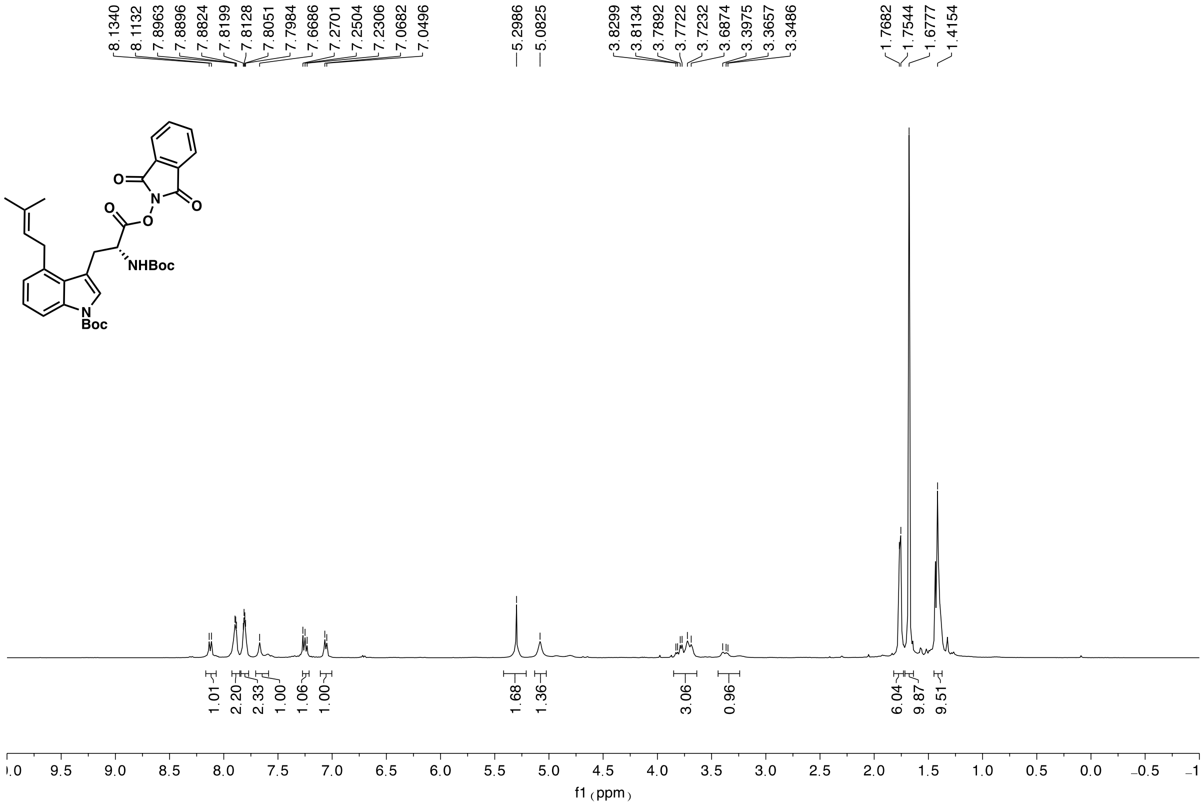

^{1}H NMR spectrum (400 MHz, $CDCl_3$) of compound 5

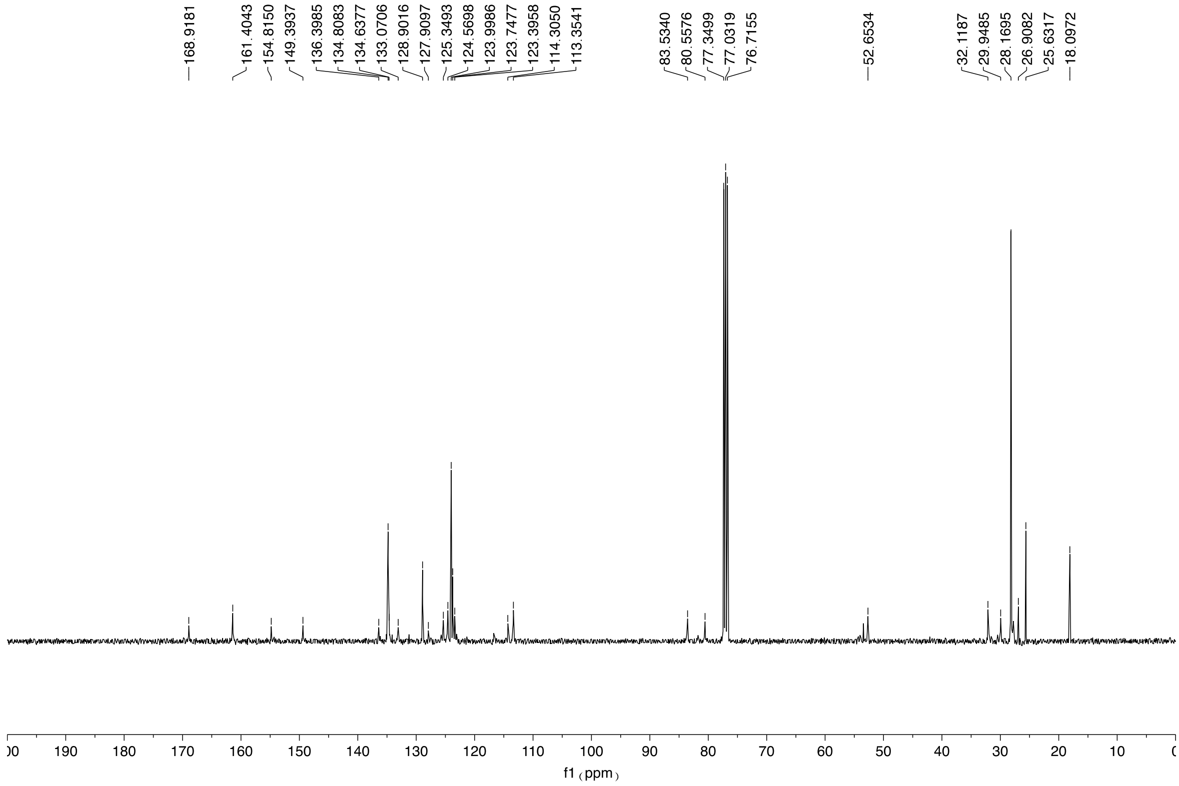

$^{13}C\{H\}$ NMR spectrum (100 MHz, $CDCl_3$) of compound 5

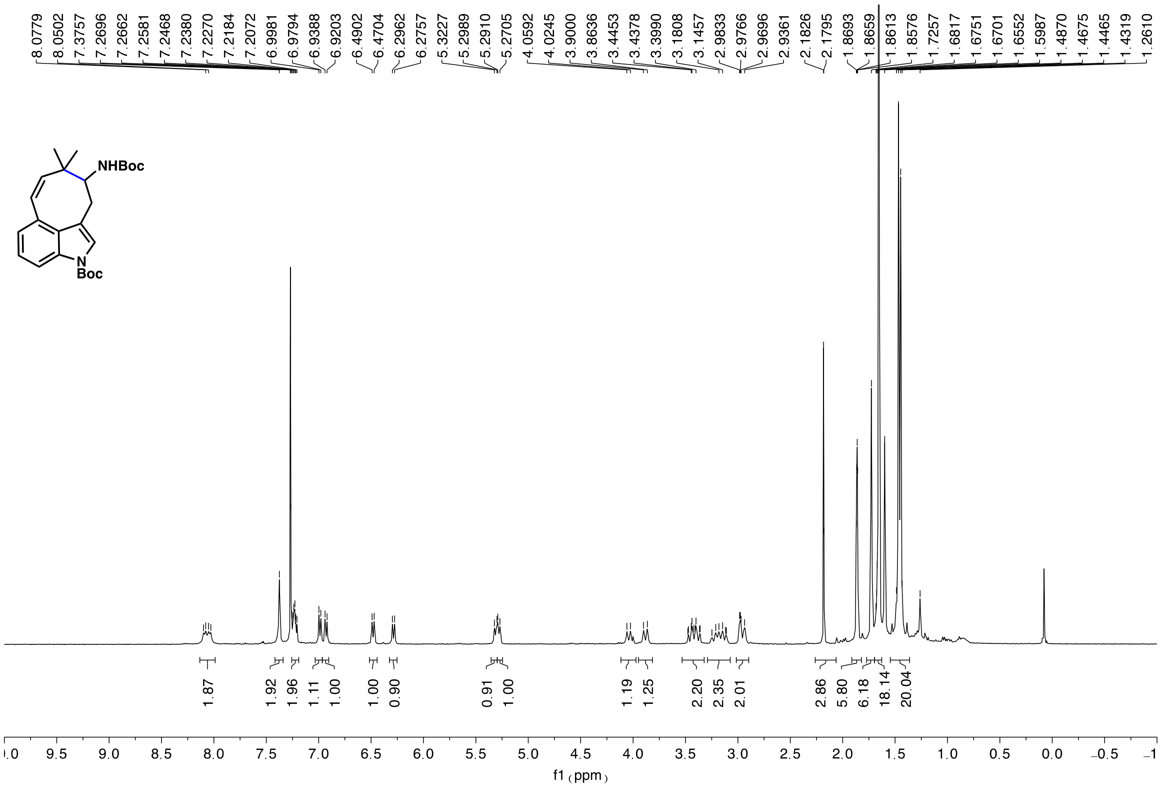

^{1}H NMR spectrum (400 MHz, $CDCl_3$) of compound 6

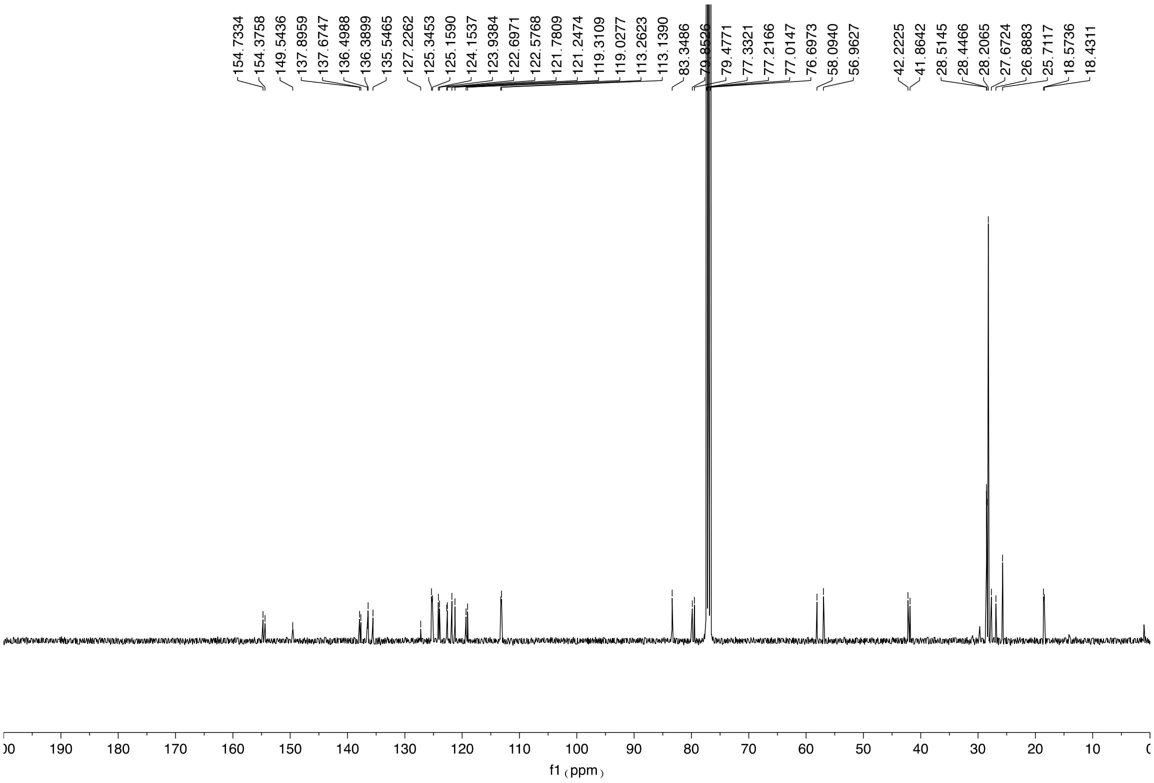

^{13}C{H} NMR spectrum (100 MHz, $CDCl_3$) of compound 6

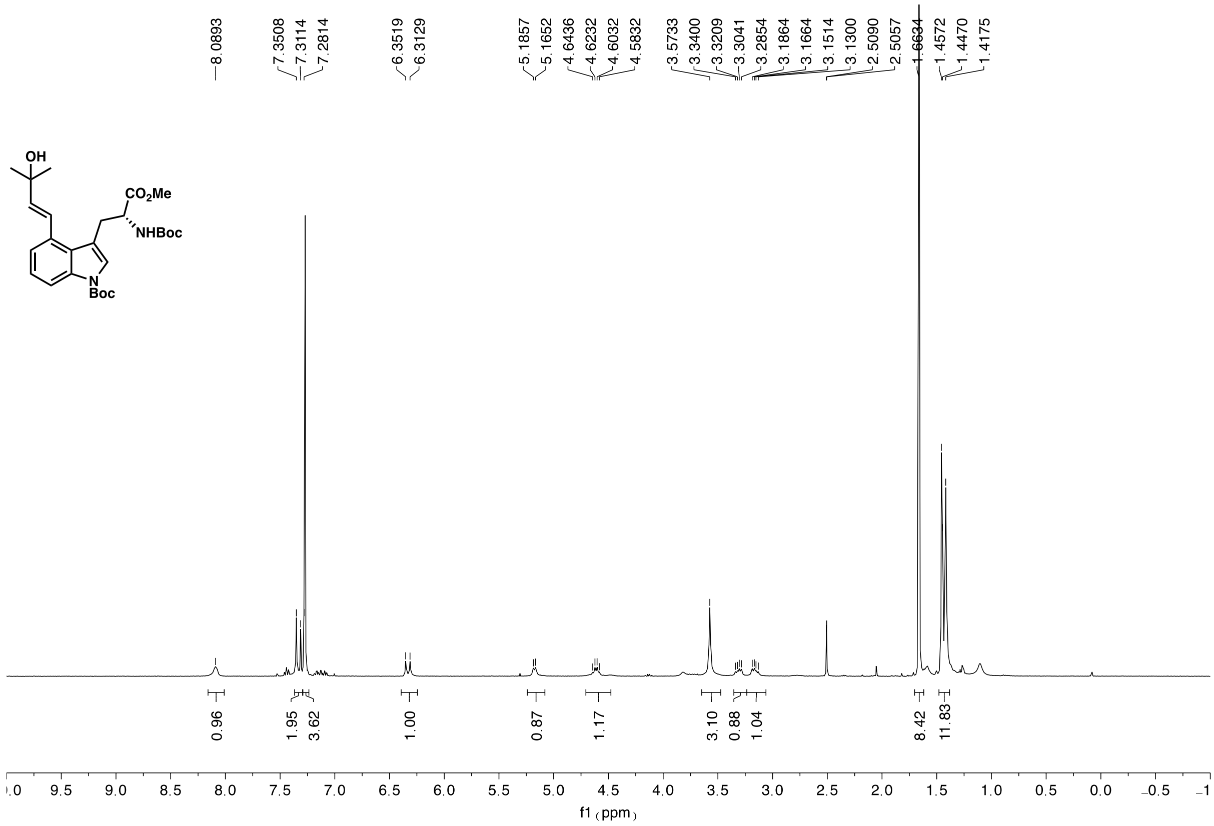

^{1}H NMR spectrum (400 MHz, $CDCl_3$) of compound 7

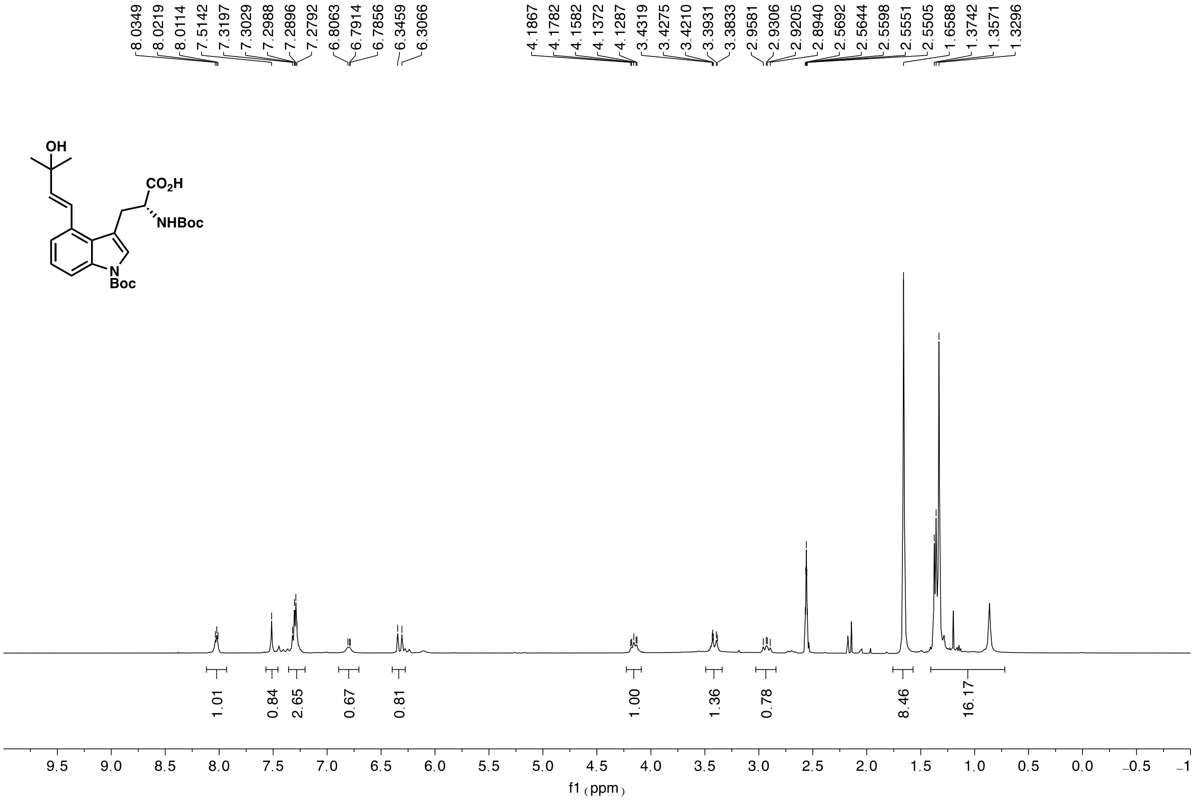

^{1}H NMR spectrum (400 MHz, DMSO-d_6) of compound 8

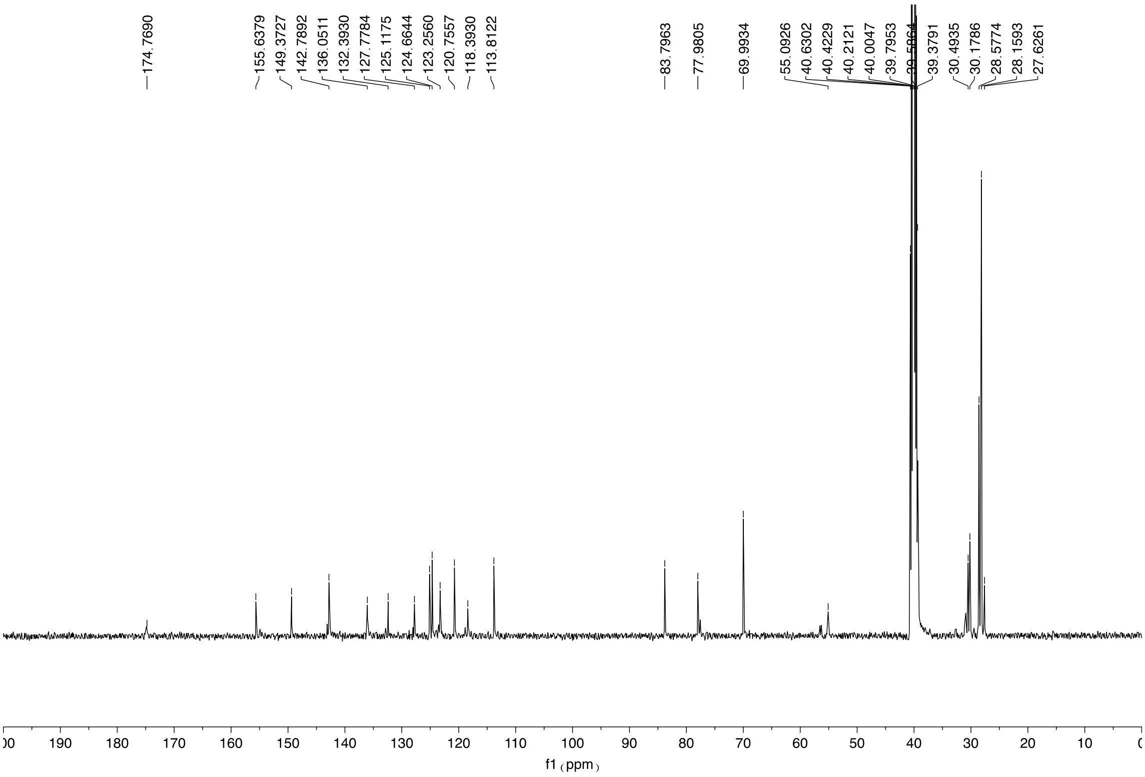

^{13}C NMR spectrum (100 MHz, DMSO-d_6) of compound 8

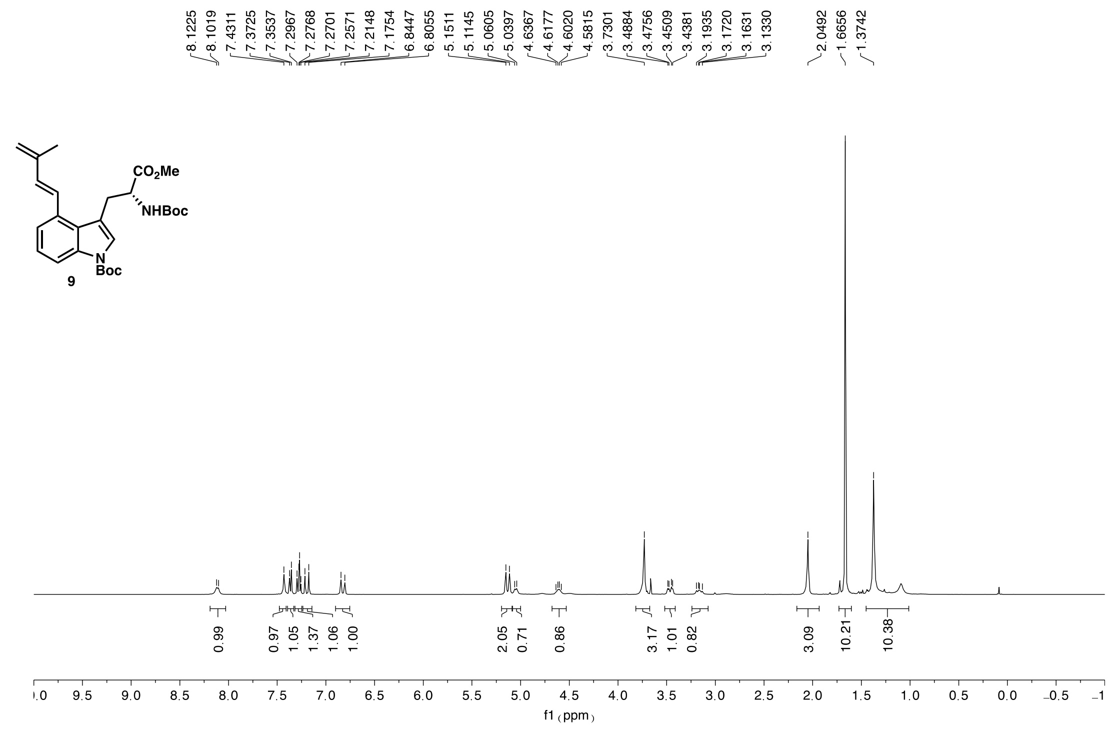

^{1}H NMR spectrum (400 MHz, $CDCl_3$) of compound 9

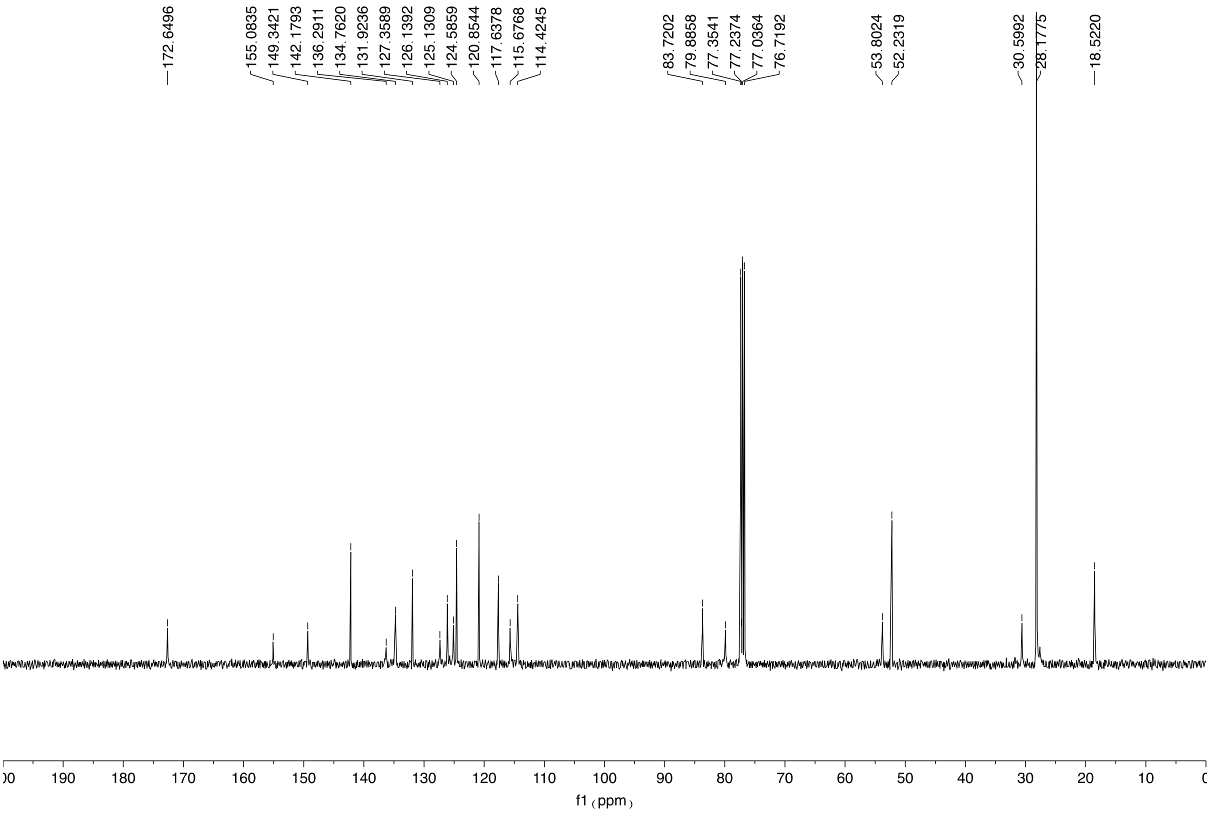

^{13}C{H} NMR spectrum (100 MHz, $CDCl_3$) of compound 9

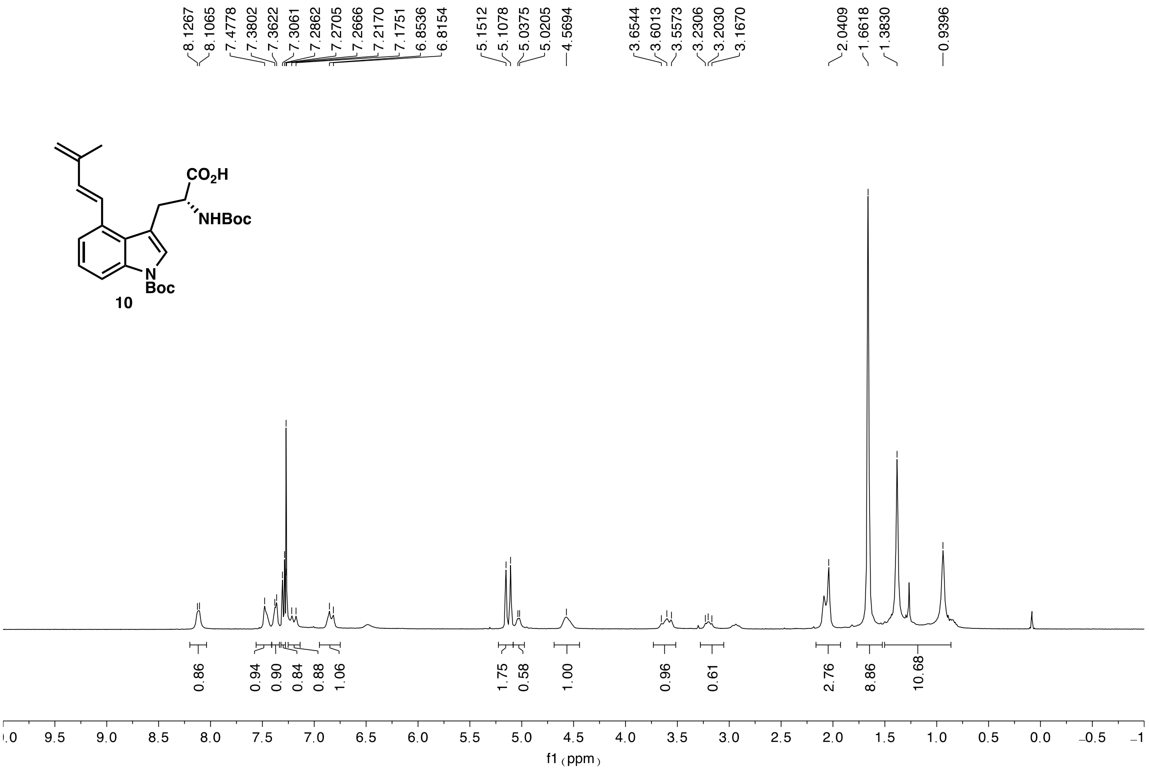

^{1}H NMR spectrum (400 MHz, $CDCl_3$) of compound 10

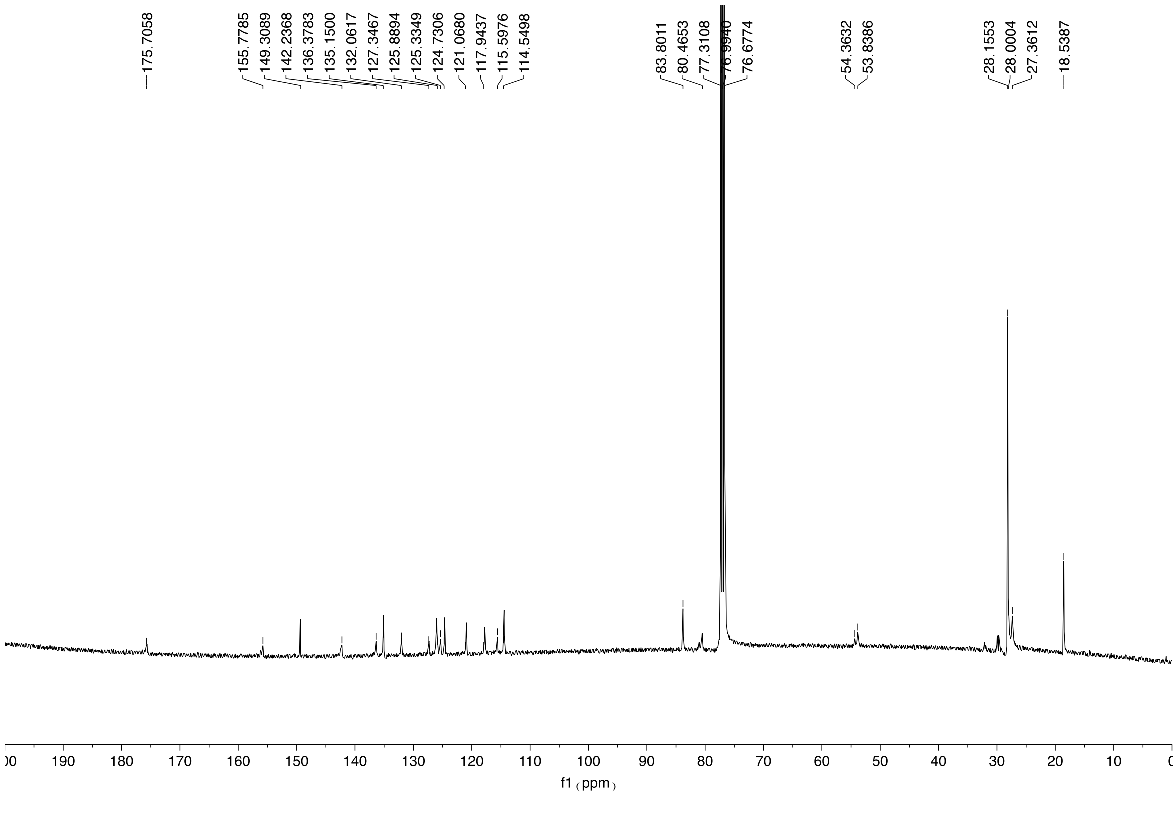

^{13}C{H} NMR spectrum (100 MHz, $CDCl_3$) of compound 10

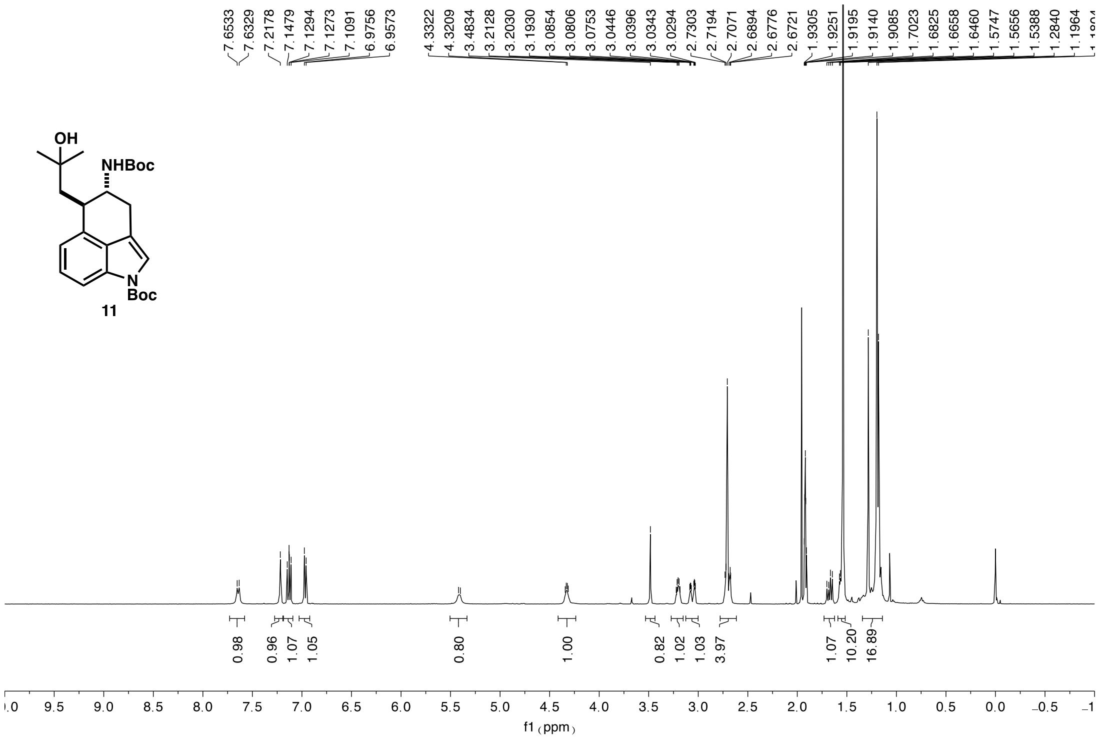

^{1}H NMR spectrum (400 MHz, Acetone-d_6) of compound 11

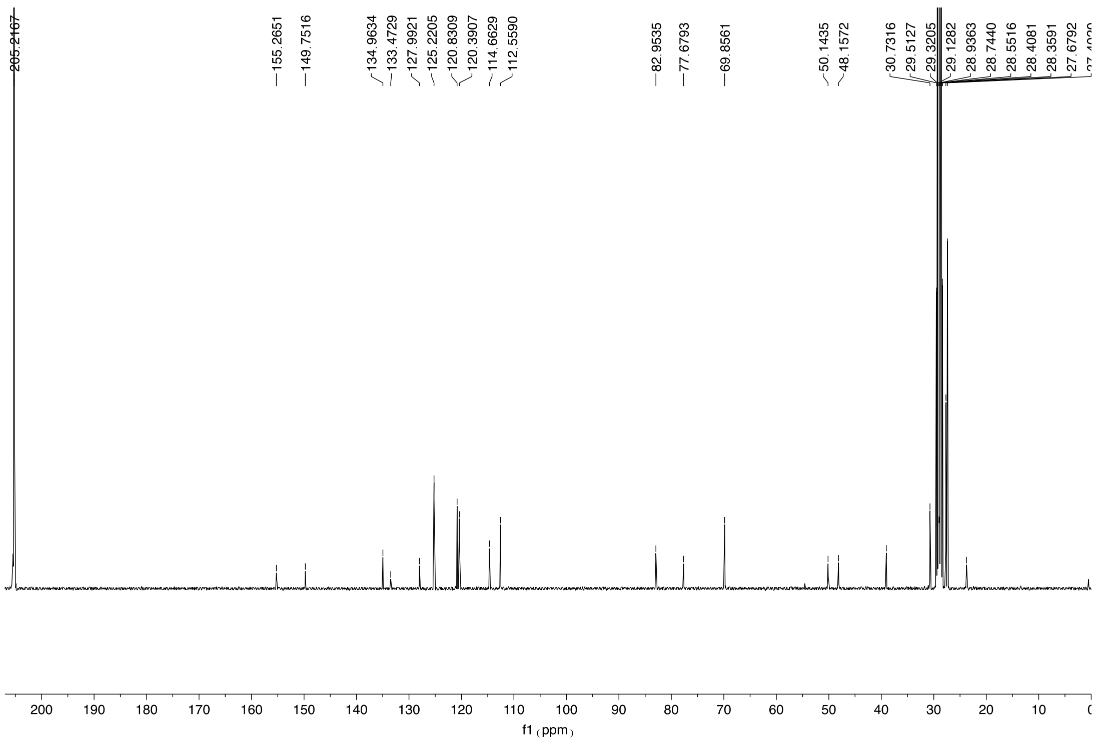

$^{13}C\{H\}$ NMR spectrum (100 MHz, Acetone-d_6) of compound 11

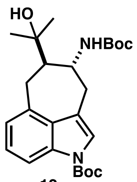

12

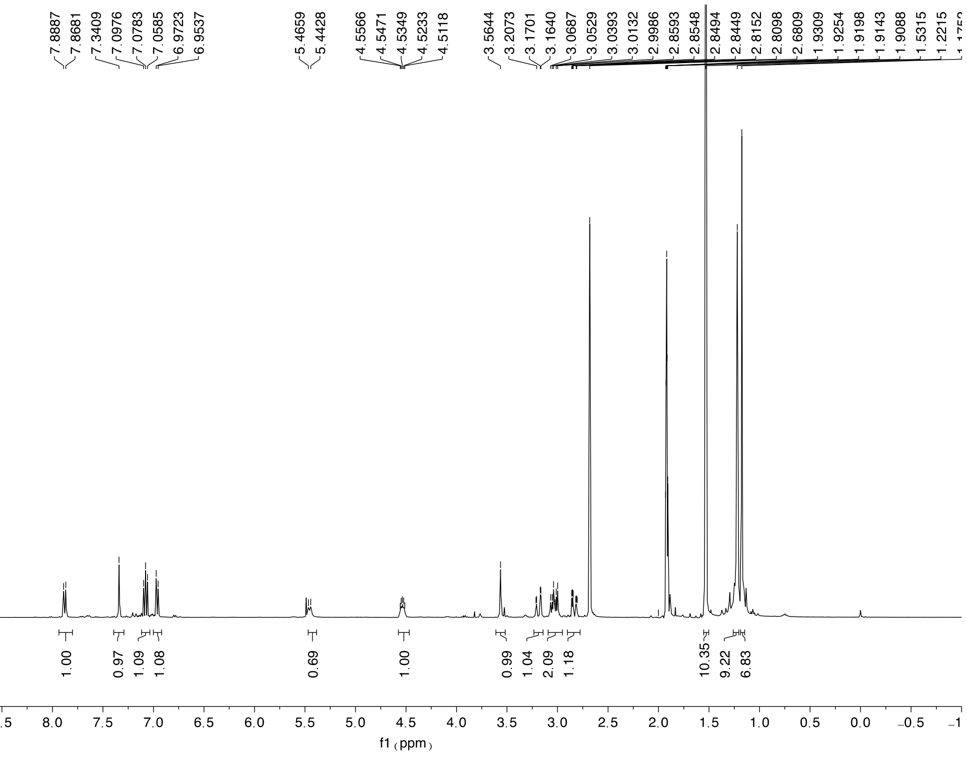

^{1}H NMR spectrum (400 MHz, CDCl$_3$) of compound 12

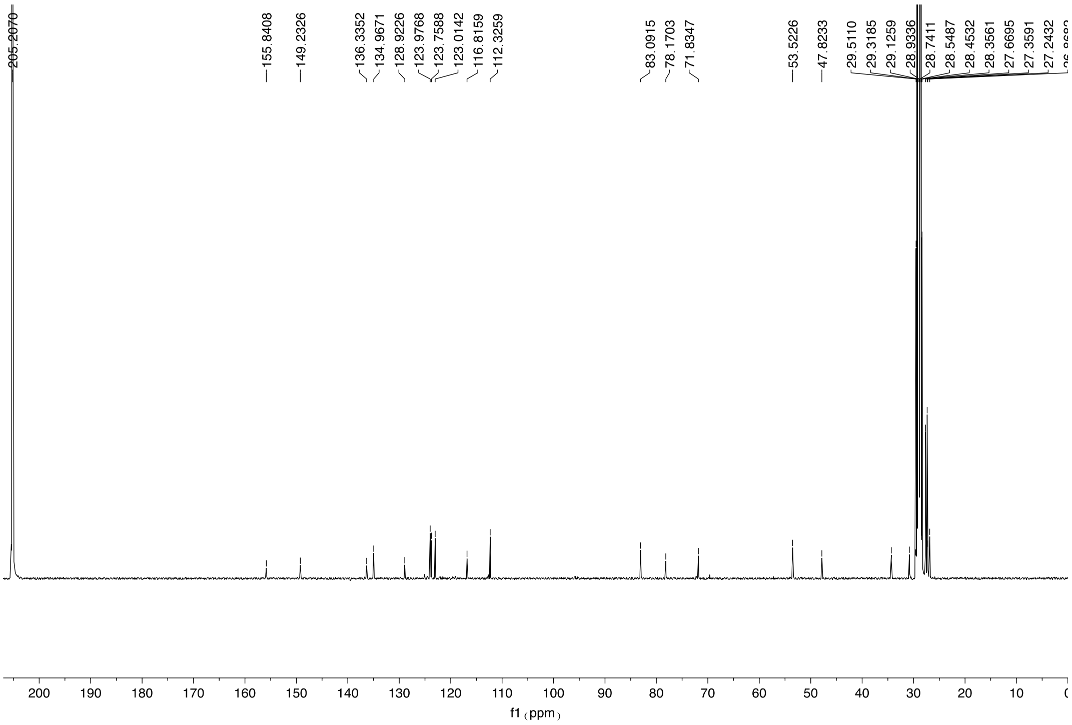

^{13}C{H} NMR spectrum (100 MHz, CDCl$_3$) of compound 12

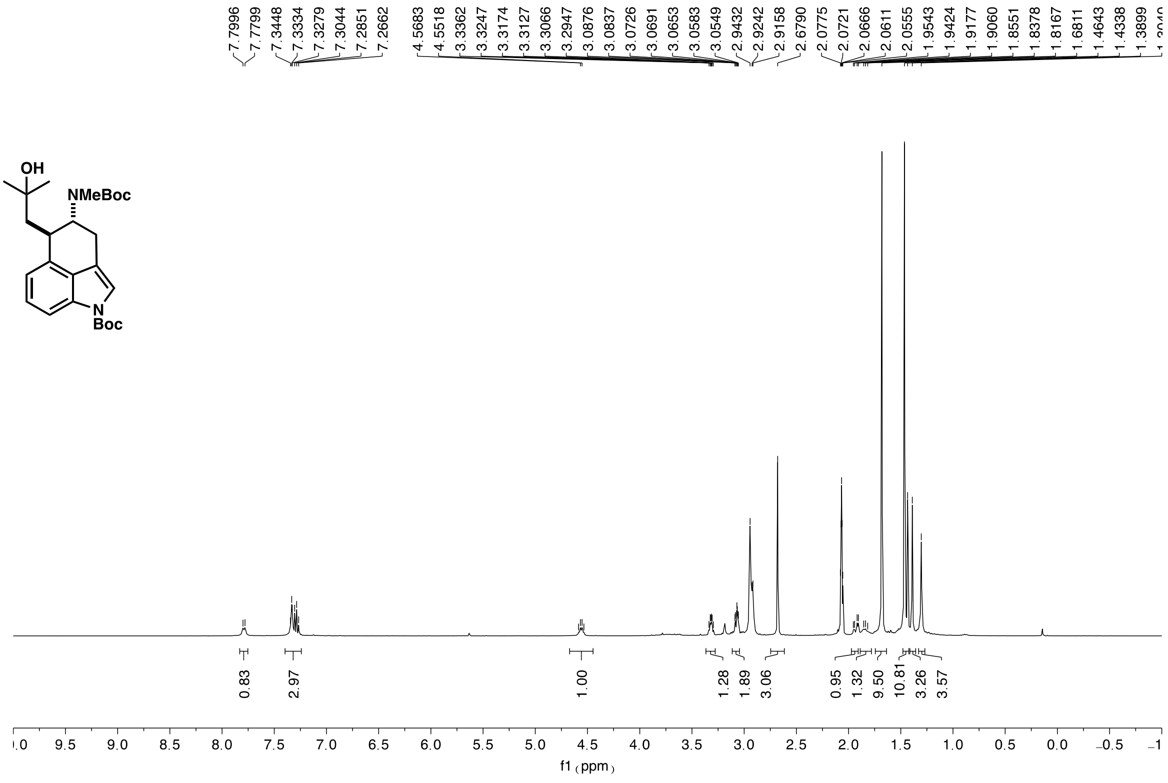

^{1}H NMR spectrum (400 MHz, Acetone-d_6) of compound 13

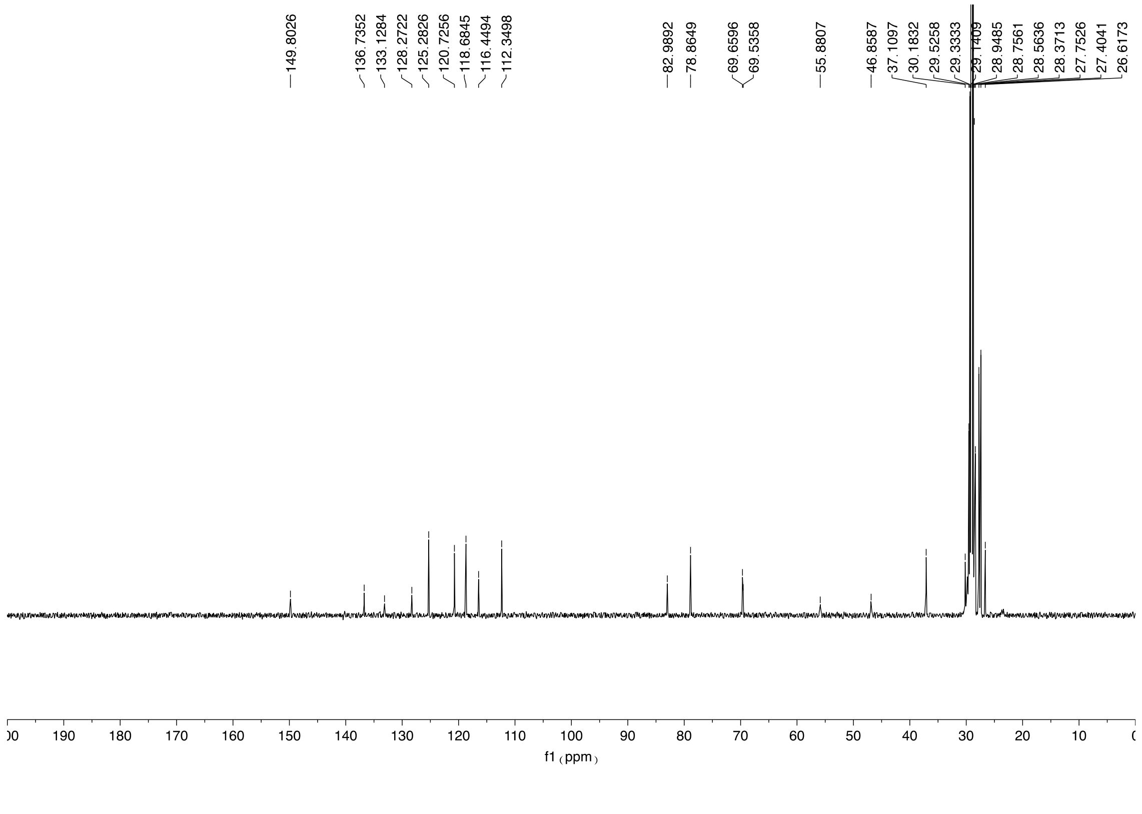

^{13}C{H} NMR spectrum (100 MHz, Acetone-d_6) of compound 13

Figure S1. Variable-temperature (VT) NMR experiments for compound **6**

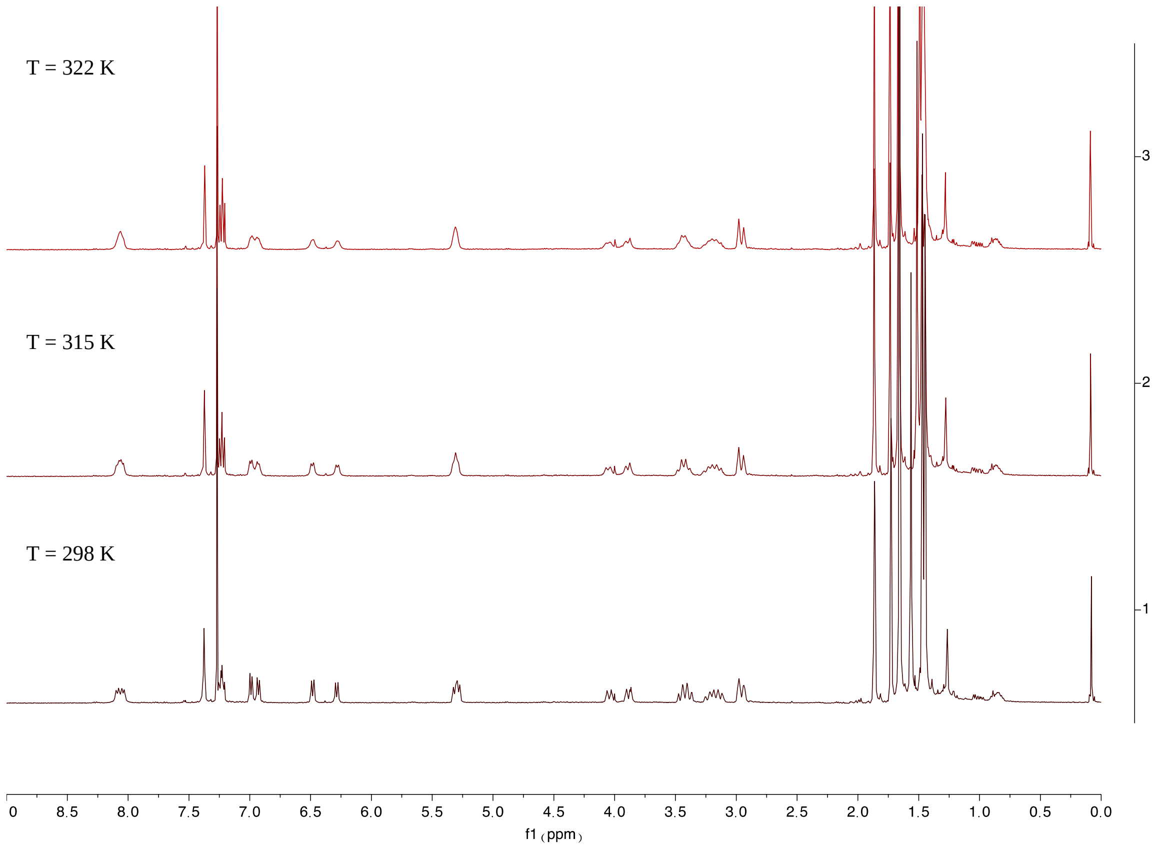

Figure S2. MS spectra of **11** and **11-*d***

Compound **11-*d*** was obtained using the same procedure of compound **11**, employing DMF-*d*7

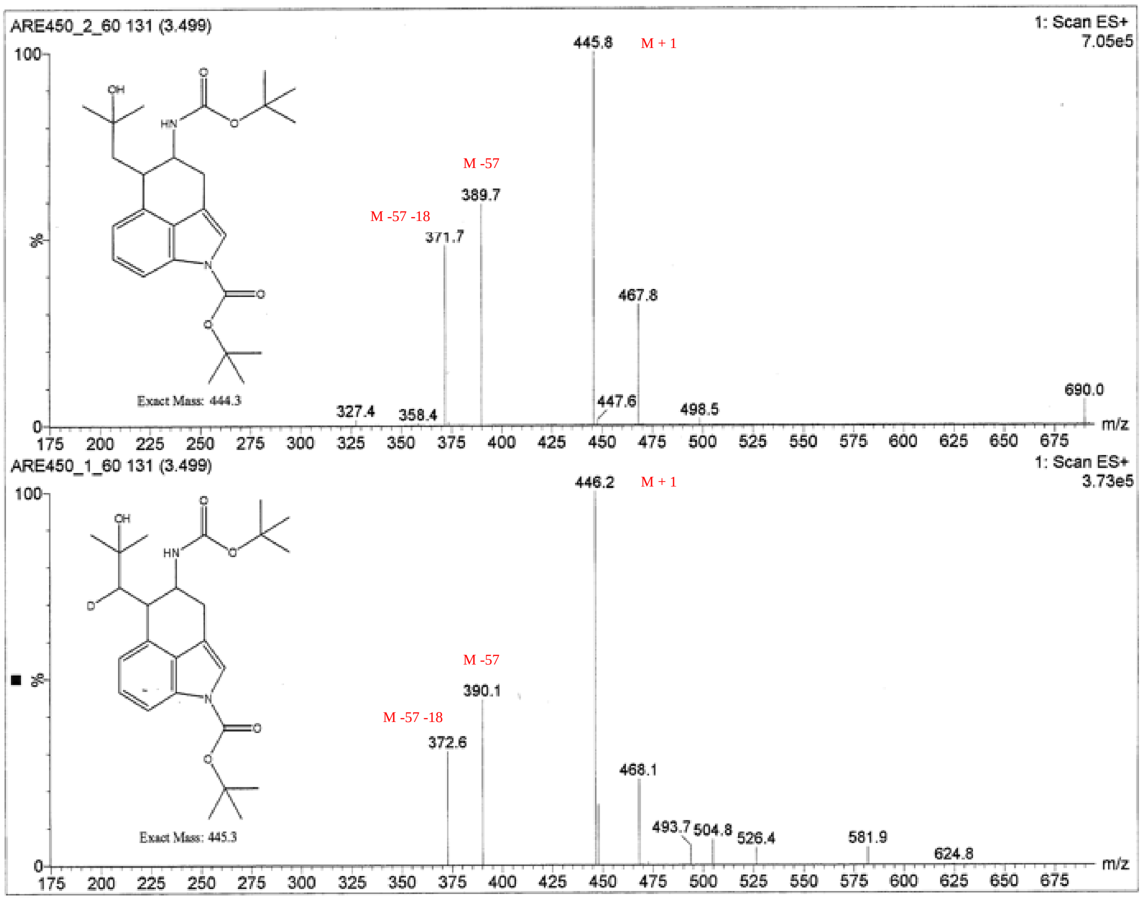

Supplement: File 1 — Experimental and copies of spectra. [file Beilstein_J_Org_Chem-19-918-s001.pdf]
